# Genetic diversity and yield trait associations in soybean germplasm using SSR markers

**DOI:** 10.1371/journal.pone.0355194

**Published:** 2026-08-04

**Authors:** Hailong Zong, Xiaolei Li, Songyin Liu, Hongchun Bao, Chongyang Bai

**Affiliations:** 1 Agricultural College, Inner Mongolia Agricultural University, Hohhot, Inner Mongolia, China; 2 Agricultural and Animal Husbandry Technology Extension Center of Inner Mongolia Autonomous Region, Hohhot, Inner Mongolia, China; Arish university, Faculty of agricultural and environmental sciences, EGYPT

## Abstract

Soybean (*Glycine max* (L.) Merrill), a vital food and oilseed crop native to China, has the development of high-yield and high-quality new varieties as a core objective in soybean breeding. However, studies on genetic diversity and marker-trait associations for yield-related traits across multiple environments using simple sequence repeat (SSR) markers remain limited. This study utilized 20 pairs of SSR primers to investigate genetic diversity and marker-trait associations (MTAs) between SSR markers and yield-related traits in 150 soybean accessions of diverse origins, which were grown under two environmental conditions. Genetic diversity analysis of 12 key agronomic traits, including plant height, number of branches, number of pods per plant, and hundred-seed weight, showed substantial phenotypic variation with large ranges and high coefficients of variation among the accessions. Cluster analysis based on agronomic traits classified the germplasm into five clusters under environment E1 and four clusters under environment E2. SSR primer amplification of the accessions’ genomic DNA produced an average of 7.10 amplified loci and 6.80 polymorphic loci per primer pair, with an average polymorphic locus rate of 96%. The average values of effective allele number (Ne), gene diversity index (H), Shannon’s information index (I), polymorphism information content (PIC), and genetic similarity coefficient (GSC) were 1.5035, 0.3581, 0.5176, 0.3539, and 0.57, respectively. SSR-based cluster analysis divided the 150 accessions into three distinct clusters. Principal coordinate analysis (PCoA) validated that the selected SSR primers could effectively discriminate among the accessions. Population structure analysis indicated complex genetic relationships and substantial genetic diversity within the germplasm panel. Association analysis between agronomic traits and SSR markers identified 44 markers significantly associated with yield-related traits, of which 13 were simultaneously linked to two or more yield-related traits. Using generalized linear model (GLM) and mixed linear model (MLM) analyses, six SSR markers significantly associated with yield-related traits were consistently detected across the two environments. These results provide a theoretical basis for germplasm utilization and molecular breeding in soybean.

## Introduction

Soybean (*Glycine max* (L.) Merrill), the world’s fourth most widely grown crop, is highly valued for its high-quality protein and vegetable oil for human and animal consumption, along with its pivotal role in nitrogen fixation during crop rotation [1]. Restricted by staple crop competition in its cropping system, China is the world’s largest soybean importer [2]. Soybean meal is the main protein source for China’s poultry industry due to its balanced amino acid. As the center of origin of soybean, China houses more than 33,000 germplasm accessions, accounting for 80% of global soybean germplasm [3]. Nevertheless, modern Chinese soybean cultivars exhibit a narrow genetic base, attributed to the heavy reliance on a limited number of parental lines, which hinders the development of elite high-yield and stress-tolerant cultivars. Therefore, broadening genetic diversity is pivotal to alleviating pest and disease risks and narrowing the yield gap [4].

Diverse genetic resources facilitate plant breeders in developing improved cultivars exhibiting desired traits. Modern cultivar development is primarily centered on identifying optimal alleles for agronomic traits, given the large number of genes governing these traits. Substantial beneficial alleles were likely lost during soybean domestication and range expansion due to genetic bottlenecks [5]. Thus, breeding accessions are required to harbor rare beneficial alleles absent in elite germplasm, making clarification of their origins critical. Genetically divergent accessions frequently harbor novel alleles, yet selecting appropriate accessions from available germplasm remains challenging [6]. Accordingly, understanding soybean genetic diversity enables breeders to grasp germplasm structure, select diverse parental lines, and expand genetic resources. Genetic diversity within and among populations is commonly evaluated using morphological characterization, biochemical markers, or molecular marker techniques [7]. Morphological and biochemical markers are less reliable than DNA markers due to their high susceptibility to environmental influences [8]. DNA markers serve as a cornerstone for investigating crop genetic diversity: they enable the detection of nucleotide sequence polymorphisms among individuals and are insensitive to environmental fluctuations [9]. Molecular markers, including Random amplified polymorphic DNA (RAPD), Simple- sequence repeats (SSR), expresses sequence tags (EST-SSR), Amplified fragment length polymorphism (AFLP), and Single nucleotide polymorphism (SNP) have been widely employed for evaluating soybean genetic diversity [10,11]. Among these, simple sequence repeat (SSR) markers are the most widely employed for gene characterization, genetic diversity analysis, and genetic linkage mapping. Their genome-wide uniform distribution, high polymorphic information content (PIC), and strong reproducibility render them ideal for genotype differentiation, pedigree analysis, genetic distance evaluation, and variety identification [12]. Single nucleotide polymorphism (SNP) markers exhibit evolutionary stability due to their low recurrent mutation rates, making them superior for dissecting the genetic basis of complex traits and exploring genomic evolution. However, SNPs are less preferred than SSRs for genetic diversity analysis, attributed to their biallelic nature, limited information content, and relatively high application costs [13]. Numerous studies have confirmed that SSR markers are effective for evaluating soybean genetic diversity and identifying marker-trait associations [14].

Yield, as a quantitative trait, represents a primary target in soybean breeding. Its expression is governed by multiple quantitative trait loci (QTLs) and modulated by natural environmental factors [15]. Comprehensive utilization and exploitation of abundant germplasm resources, coupled with in-depth investigations into soybean yield-related traits, are pivotal to breeding high-yielding and high-quality soybean cultivars and ensuring soybean supply security [16]. Accordingly, this study employed 150 soybean germplasm accessions of diverse origins for field trials under two distinct environmental conditions. Phenotypic characterization of 12 yield-related traits was performed, followed by genotyping with SSR markers. Association analysis between SSR markers and phenotypic data of yield-related traits uncovered SSR loci tightly associated with these traits. This study lays a theoretical basis for molecular marker-assisted selection (MAS) in soybean breeding, as well as for the conservation and exploitation of soybean germplasm resources.

## Materials and methods

### Plant materials

A total of 150 soybean germplasm accessions with diverse geographic origins were employed in this study (S1 Table), and field experiments were conducted at two experimental sites affiliated with Inner Mongolia Agricultural University, Hohhot, Inner Mongolia Autonomous Region. The field and climatic conditions of the two sites during the 2024 growing season are detailed as follows: E1 was located at Hailiutu Science and Technology Park (40°68′N, 111°37′E), characterized by sandy loam soil, with a mean temperature of 18.5 ℃, annual precipitation of 420 mm, and relative humidity of 60% during the growing period. In contrast, E2 was situated at East Science and Technology Park (40°81′N, 111°72′E); while it also had sandy loam soil, its climatic conditions were slightly less favorable, with a mean temperature of 17.8 ℃, annual precipitation of 390 mm, and relative humidity of 58% during the same growing season. A randomized complete block design with three replications per accession was adopted, and plots were surrounded by protective rows; irrigation was provided via a sprinkler system. Sowing was conducted in May 2024 and harvesting in October 2024, with the following planting parameters: 2 m long rows, 50 cm row spacing, 10 cm plant spacing, 1 m inter-row aisles, and three rows per accession. Field management practices were consistent with commercial agricultural practices. In June 2024, young leaves were sampled from the 150 soybean materials described above. These samples were used for DNA extraction and SSR molecular marker detection. Several tender leaves were taken from each material, wrapped with labeled aluminum foil, and transported in fully submerged liquid nitrogen. After being delivered to the lab, they were quickly preserved in an ultra-low temperature freezer.

### Phenotypic data

In October 2024, 3 plants were randomly selected from each plot in both environments to assess 12 key agronomic traits, for which the detailed measurement protocols are described as follows: Plant height was defined as the distance from the cotyledon node to the apex of the main stem, measured using a ruler with the unit of cm; pod base height was determined as the distance from the cotyledon node to the first pod with the unit of cm; the number of main stem nodes was counted manually as the total number of nodes on the main stem; the number of pods per plant was defined as the total number of effective pods per plant, determined by manual counting; stem diameter was measured at 10 cm above the cotyledon node using a vernier caliper with the unit of mm; pod length and width were calculated as the average values of 10 randomly selected pods, with units of cm and mm respectively; the number of grains per plant was obtained via manual counting of the total grains per plant; seed weight per plant was weighed using an electronic balance with an accuracy of 0.01 g and the unit of g; hundred-seed weight was determined as the average weight of three replicate samples with 100 grains per replicate, measured with an electronic balance with the unit of g; and grain length and width were recorded as the average values of 10 randomly selected grains, with both parameters expressed in mm.

### DNA extraction and SSR genotyping

Total genomic DNA was extracted from samples using the Plant Genomic DNA Kit (TianGen, Beijing, China). DNA quality and concentration were initially assessed by 1% agarose gel electrophoresis, while DNA concentration and purity were subsequently quantified using a NanoDrop 2000 spectrophotometer (Thermo Fisher Scientific, Wilmington, DE, USA). All DNA samples were diluted to a uniform concentration of 20 ng/μL in TB elution buffer (TianGen) and aliquoted, then immediately stored at −80°C for subsequent PCR amplification.

Twenty pairs of SSR primers with high polymorphism were screened and synthesized by Shanghai Sangon Biotech Co., Ltd. (Shanghai, China) (S2 Table in S1 File). The primer sequences were numbered uniformly from A to T to correspond to the 20 primer pairs. The markers were selected on account of their uniform distribution across the soybean genome and superior amplification performance, with the underlying assumption that 10–30 highly polymorphic markers would be sufficient to generate high-quality and accurate data [17]. These 20 SSR primer pairs were used for PCR amplification of genomic DNA from 150 test samples.

Polymerase chain reaction (PCR) amplification was carried out in a total volume of 20 μL, consisting of 2 μL genomic DNA (20 ng/μL), 0.9 μL forward primer (10 μM), 0.9 μL reverse primer (10 μM), 0.1 μL Taq DNA polymerase (5 U/μL), 2 μL Mg^2+^ -containing buffer (2 mM), 1.4 μL dNTPs (2.5 μM), and 12.7 μL ddH_2_O. PCR was performed on a BIO-RAD T100 Thermal Cycler (Bio-Rad Laboratories, Hercules, CA, USA) following a previously reported protocol, with the program as follows: an initial denaturation at 94 °C for 5 min; followed by 20 touchdown cycles of 94 °C for 30 s, 40.3–60.5 °C (decreasing by 0.5 °C per cycle) for 30 s, and 72 °C for 1 min; then 20 additional cycles of 94 °C for 30 s, and 72 °C for 45 s; and a final extension at 72 °C for 10 min. Polymorphisms of the SSR primers were visualized using 8% polyacrylamide gel electrophoresis.

### Data analysis

Data for 12 key agronomic traits of 150 test materials grown under two cultivation environments were organized using Microsoft Excel 2021 software. Descriptive statistics were conducted to calculate the minimum, maximum, mean, standard deviation (SD), and coefficient of variation (CV) for each trait. Genetic diversity index calculation: Shannon’s information index (H′), H′ = -ΣPi × ln Pi, was used for the genetic diversity of each trait, where Pi represents the percentage of the i-th grade trait in the total number of copies. Phenotypic data from the two environments were analyzed separately.

SSR-PCR band analysis and data processing were performed using a D100 DNA marker as the reference standard for relative molecular weight determination. Amplified bands from each pair of polymorphic primers were recorded quantitatively. For each primer pair, the presence or absence of DNA bands at the same migration position on the gel was scored: bands present were designated as “1,” and absent bands as “0.” These data were imported into Microsoft Excel to construct a binary (0/1) data matrix.

Using the marker-derived binary matrix, PopGene 32 was employed to estimate the effective number of alleles (Ne), Shannon’s information index (I), and gene diversity index (H) for the 150 accessions. PowerMarker was used to calculate the polymorphism information content (PIC). The genetic similarity coefficient (GSC) among accessions was estimated using NTSYS-pc 2.10e, from which a genetic similarity matrix (SM) was constructed. Subsequently, frequency statistics were performed with an interval size of 0.02 to generate a frequency distribution histogram of GSC values. Principal Coordinate Analysis (PCoA) was conducted in R 4.4.2 based on Bray-Curtis dissimilarity coefficients. An unweighted pair group method with arithmetic mean (UPGMA) phylogenetic tree was constructed using Tassel 5 and visualized via the online tool iTOL v7 (https://itol.embl.de/). Population structure analysis was performed in STRUCTURE 2.3.4 under a Bayesian framework, with five independent simulations conducted for each K value (K = 2–10). The burn-in iterations were set to 10,000, and Markov chain Monte Carlo (MCMC) iterations were set to 100,000. The optimal K value was identified using the online tool Structure Selector (https://lmme.ac.cn/StructureSelector/index.html). The CLUMPP 1.1.2 software processes data from 10 independent runs to determine the best K value, while also calculating the Q value. Associative analysis was performed to explore the relationships between 20 pairs of SSR molecular markers and 12 key agronomic traits under two distinct cultivation environments, utilizing generalized linear models (GLMs) and mixed linear models (MLMs) implemented in Tassel 5 software. To reduce false associations caused by population stratification and genetic relatedness, we included the Q matrix from population structure analysis and the K matrix from SSR genotyping. The GLM model used only the Q matrix to correct for population structure. The MLM model used both the Q matrix to control for population structure and the K matrix to account for genetic relatedness between accessions. Polymorphic SSR marker data were treated as explanatory variables, whereas phenotypic data of agronomic traits served as response variables. Marker-trait associations were considered statistically significant when the probability value met *P* < 0.05. Phenotypic variation explained (PVE) indicated the fixed marker effects. Subsequently, Manhattan plots were constructed to visually illustrate the outcomes of the associative analysis.

## Results

### Phenotypic diversity

Across the two cultivation environments (E1 and E2), the test materials displayed high variability in most of the 12 key agronomic traits, while exhibiting low dispersion in the remaining few (S3 Table in S1 File). The traits with high variability were concentrated on grains per plant, pods per plant, seed weight per plant, and stem diameter. The coefficients of variation (CVs) for these four traits were significantly higher than those of the other traits, with their corresponding genetic diversity indices also being relatively elevated. The remaining eight traits, including main stem node number, pod base height, pod width, plant height, hundred-seed weight, seed length, seed width, and pod length, showed lower dispersion: their CVs were generally below 20%, and their genetic diversity indices were relatively moderate. Minor inter-environmental differences were observed: in E1, the trait with the highest CV was grains per plant (34.77%), followed by pods per plant (34.63%); in E2, the trait with the highest CV was pods per plant (36.94%), followed by grains per plant (36.75%). Notably, the CVs of these four highly variable traits were slightly higher in E2 than in E1 overall.

### Phenotypic trait cluster analysis

**Fig 1:**
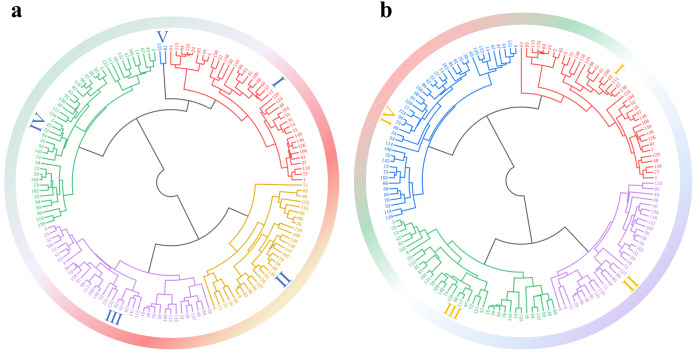
UPGMA clustering of agronomic traits of test materials in (A) E1, (B) E2.

Cluster analysis was conducted based on phenotypic data of 12 key agronomic traits from 150 test accessions grown under E1 and E2 environments (Fig 1; S4 Table in S1 File). In the E1 environment, the accessions were grouped into 5 clusters: Cluster I (38 accessions) exhibited significantly superior pod base height compared to the other 4 clusters; Cluster II (30 accessions) showed a distinct advantage in seed length and could be used as candidate parents for improving grain appearance and marketability; Cluster III (37 accessions) excelled in four yield-related traits, namely plant height, main stem node number, pods per plant, and grains per plant, and among these accessions, accessions 59 and 28 had notably higher plant heights (86.88 cm and 86.86 cm, respectively) than the other accessions in this cluster; Cluster IV (43 accessions, the largest cluster) displayed significantly superior pod width; Cluster V (2 accessions: 70 and 122) had significantly superior hundred-seed weight and plant heights significantly lower than the overall mean, aligning with the characteristics of dwarf high-yielding germplasm and making them suitable as parents for dwarfing breeding.

In the E2 environment, the accessions were classified into 4 clusters: Cluster I (38 accessions) exhibited significant advantages in both pod base height and hundred-seed weight; Cluster II (31 accessions) showed distinct advantages in seed length and seed width, thereby providing excellent germplasm resources for improving soybean seed morphology; Cluster III (37 accessions) excelled in four yield-component traits, including pods per plant, pod length, grains per plant, and seed weight per plant, and accession 15, in particular, demonstrated outstanding performance with 130 grains per plant and a total seed weight of 34.51 g per plant, rendering it suitable for selection as a parent for high-yielding germplasm development; Cluster IV (44 accessions) displayed excellent pod width.

### Genetic diversity of soybean based on SSR markers

**Fig 2:**
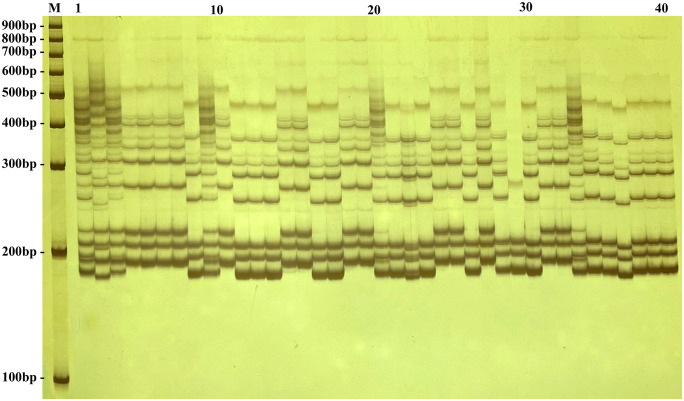
Amplification electropherogram of primer Sat_196. The horizontal axis denotes materials numbered 1-40, while the vertical axis represents the amplification range of Sat_196.

A total of 150 test materials were genotyped using 20 pairs of SSR primers with high polymorphism (Fig 2). PCR amplification yielded 142 loci in total, of which 135 were polymorphic (S5 Table in S1 File). The average percentage of polymorphic loci was 96%, with a mean of 7.10 amplified loci and 6.80 polymorphic loci per primer pair. Among the 20 SSR primer pairs, Sat_196 and Satt316 produced the highest number of polymorphic loci (9 per primer pair), while Sat_258 and Satt046 produced the lowest (4 per primer pair). Genetic diversity analysis based on the SSR data showed the following: the effective number of alleles (Ne) ranged from 1.2181 to 1.6902 (mean = 1.5035); the gene diversity index (H) ranged from 0.2723 to 0.4251 (mean = 0.3581); the Shannon’s information index (I) ranged from 0.4207 to 0.6365 (mean = 0.5176); and the polymorphic information content (PIC) ranged from 0.2702 to 0.4198 (mean = 0.3539). These findings demonstrate that the test lines possess abundant genetic diversity.

### Genetic similarity coefficient and cluster analysis

**Fig 3:**
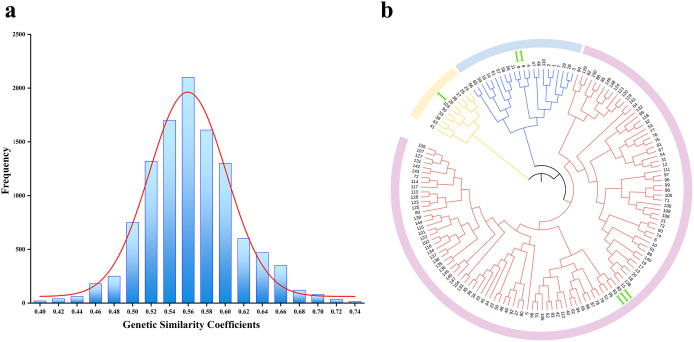
Genetic Similarity Coefficient and Cluster Analysis. (a) Frequency distribution of genetic similarity coefficients. (b) UPGMA clustering results of SSR molecular markers.

Based on 142 marker loci detected by 20 pairs of polymorphic SSR primers, genetic similarity coefficients (GSC) were calculated among 150 test accessions (Fig 3a). The frequency distribution of GSCs across all test accessions exhibited a unimodal pattern, with values ranging from 0.40 to 0.74 and a mean of 0.57, and these results indicate extensive genetic diversity among the accessions.

Cluster analysis was performed via the unweighted pair-group method with arithmetic mean (UPGMA) (Fig 3b), which classified the 150 test accessions into three distinct clusters: Cluster I (11 accessions, accounting for 8% of the total) consisted entirely of accessions from Heilongjiang; Cluster II (22 accessions, 14% of the total) included 12 accessions from Heilongjiang and 10 from Beijing; and Cluster III (117 accessions, 78% of the total) comprised 80 accessions from Heilongjiang, 22 from Beijing, and 15 from Inner Mongolia. Notably, most accessions from the same geographical origin clustered together, suggesting relatively closer genetic relationships among accessions sharing the same origin. This indicates that the clustering results exhibited a correlation with geographical origin.

### Principal component analysis

**Fig 4:**
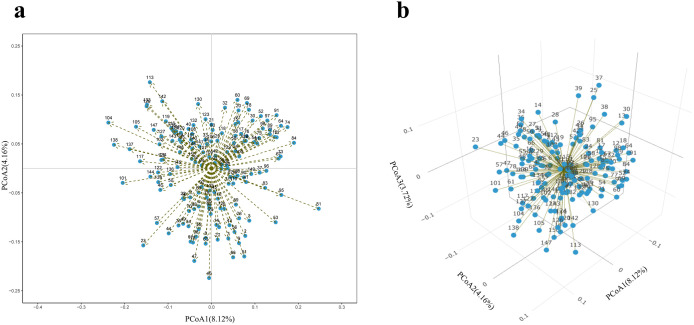
Principal Component Analysis. (a) Two-dimensional principal coordinate chart. (b) Three-dimensional principal coordinate chart. The first, second, and third principal coordinates explained 8.12%, 4.16%, and 3.72% of the total genetic variation, respectively.

Principal coordinate analysis (PCoA) was performed based on the genetic similarity among 150 test materials, using data from 20 polymorphic SSR primer pairs (Fig 4). In the two-dimensional PCoA plot, all test materials were widely distributed across four distinct groups in the space of the first two principal coordinates (PCoA1 and PCoA2), which demonstrated that the 20 selected SSR primer pairs effectively discriminated among the 150 test materials. Based on their positions along PCoA1 and PCoA2, the test materials could be categorized into four distinct groups, with clear separation between these groups. The three-dimensional PCoA plot extended the two-dimensional classification by integrating the third principal coordinate (PCoA3), and it more comprehensively and intuitively visualized the genetic differentiation among all test materials.

### Population structure analysis

**Fig 5:**
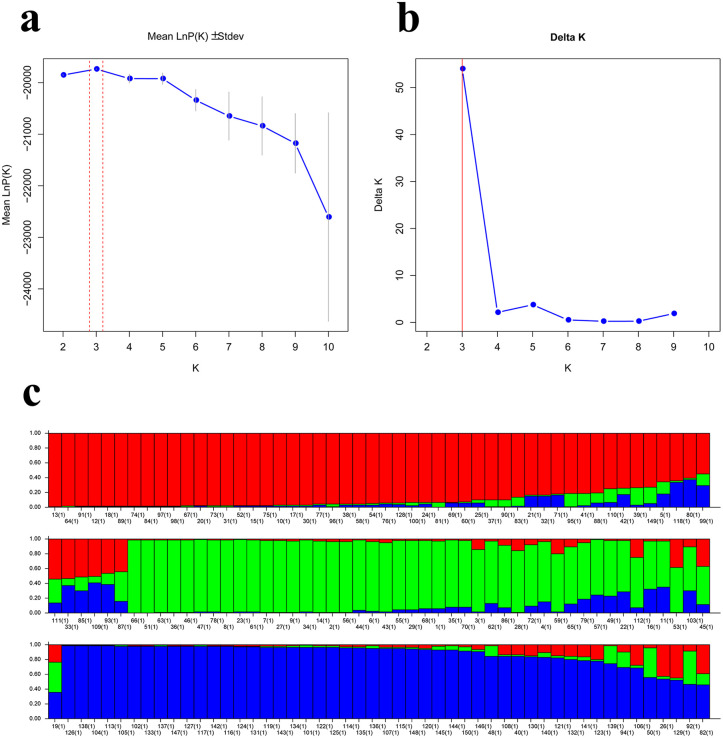
Population structure analysis of 150 soybean germplasms. (A) Scatter plot of the mean L(K) (±SD) for each K value under five replicates. (B) Delta K showing the number of populations. (C) Bar plot of populations sorted by kinship matrix.

Population genetic structure of the 150 soybean germplasms was characterized using genotypic data generated from 20 polymorphic SSR primer pairs (Fig 5). Model-based clustering revealed a continuous increase in the log-likelihood value with rising K, whereas the ΔK statistic peaked sharply at K = 3, suggesting the tested panel was optimally partitioned into three distinct subpopulations (Table 1). In total, 134 germplasms were assigned to discrete subpopulations with Q > 0.6 (S6 Tables in S1 File). Specifically, 44 germplasms belonged to Subpopulation 1, 42 to Subpopulation 2, and the remaining 48 to Subpopulation 3. The other 16 germplasms displayed Q values below 0.5 across all three inferred subgroups without predominant genetic ancestry, and were consequently categorized as admixed germplasm.

**Table 1:** Group distribution of 150 soybean germplasms based on population structure.

Province	Group1	Group2	Group3	Mixed group
Beijing	11	12	10	2
Heilongjiang	22	30	37	14
Inner Mongolia	14	0	1	0
Total	44	42	48	16

### Association Analysis of Important Agronomic Traits with SSR Molecular Markers

Association analysis between 12 key agronomic traits and SSR molecular markers was performed using generalized linear models (GLM) and mixed linear models (MLM) under two cultivation environments (E1, E2). The 12 agronomic traits included plant height, pod base height, main stem node number, pods per plant, stem diameter, pod length, pod width, grains per plant, seed weight per plant, hundred-seed weight, seed length, and seed width, with corresponding results summarized in Supplementary Figures 1-12 in S2 File and Supplementary Tables S7-S18 in S1 File. Across the two environments, 8–18 significantly associated SSR marker loci were detected per trait, with the highest number of loci (18) observed for hundred-seed weight and the lowest (8 each) for plant height and seed width. Overall, more associated loci were detected in E1 than in E2; for example, 5 loci were detected for plant height in E1 versus 3 in E2, and 8 loci for pod base height in E1 versus 4 in E2. Regarding model detection characteristics, some loci were consistently detected by both GLM and MLM, such as Sat_196 and Satt302 for plant height in E1, and Sat_148 and Satt282 for pod base height in E1. In contrast, GLM identified more unique loci, including Satt282 for plant height in both E1 and E2 and Satt302 and Satt278 for main stem node number in E1, whereas MLM detected far fewer unique loci, such as Sat_320 for pod base height in E1 and Satt220 for stem diameter in E2.

The percentage of phenotypic variation explained (PVE) by all associated loci ranged from 2.46% (pod width in E2) to 6.21% (grains per plant in E2), among which marker Satt436 exhibited particularly prominent performance: it explained 6.21% of the phenotypic variation for grains per plant in E2, 5.66% for main stem node number in E1, and over 4.5% for seed weight per plant in both environments. Additionally, markers such as Sat_148 (5.46% PVE for pod base height in E1) and Satt005 (over 4.4% PVE for hundred-seed weight in both E1 and E2) also showed relatively high PVE values. Notably, several markers were consistently detected by both models across the two environments, exhibiting stable trait associations: these included Satt592 (associated with pod width), Satt220 and Satt278 (associated with grains per plant), Satt302 (associated with seed weight per plant), and Satt005 and Satt316 (associated with hundred-seed weight).

## Discussion

Phenotypic diversity is essential for crop improvement and germplasm utilization, laying a basis for phenotypic adaptation research [18]. In this study, 150 soybean accessions were phenotyped for 12 agronomic traits across two environments (E1 and E2), and phenotypic diversity was quantified via the Shannon‑Weaver index (H′). The average H′ values were 1.80 (E1) and 1.82 (E2), revealing abundant phenotypic variation across the tested panel [19]. As summarized in Table S3 in S1 File, the coefficient of variation (CV) and H′ did not follow identical changing trends across measured traits: for E1, grain number per plant had the maximum CV (34.77%) but not the highest H′, while pod number per plant possessed the largest H′ (2.11) without the peak CV value [20]. This discrepancy confirms that CV and H′ characterize different dimensions of phenotypic variation; accordingly, the two parameters are jointly applied to comprehensively evaluate phenotypic divergence in most related studies [21]. Overall, both CV and H′ values were comparatively high in our dataset, demonstrating extensive phenotypic differentiation among the examined soybean germplasm.

Cluster analysis based on the 12 agronomic traits divided accessions into 4–5 clusters across the two environments (Fig 1). Partial overlap among germplasm clusters was observed, and germplasm from the same provenance did not cluster entirely together. This result is consistent with previous findings in soybean (*Glycine max*) and wheat (*Triticum aestivum*) [22,23]. This phenomenon is presumably due to the fact that quantitative traits are generally controlled by multiple genes with minor effects and are highly sensitive to genotype-by-environment (G×E) interactions [24]. Inherently, this study has certain limitations, given its reliance on data from a single year and limited geographic locations, which may not fully capture environmental variability or long-term trends. Future studies incorporating data across multiple temporal and spatial scales will improve the applicability and generalizability of the findings.

Soybean genetic diversity is essential for yield improvement. SSR markers have been widely used to evaluate genetic diversity in soybean germplasm [25,26]. Previous research analyzed genetic diversity in soybean varieties using SSR primer pairs, revealing an average polymorphism information content (PIC) of 0.3357 and an average gene diversity index of 0.4121, indicating rich genetic diversity in the tested materials [27]. Twenty SSR primer pairs generated a total of 142 amplified loci, 135 of which were polymorphic. The average proportion of polymorphic loci was 96%, with mean effective number of alleles (Ne = 1.5035), gene diversity (H = 0.3581), Shannon’s information index (I = 0.5176), and polymorphism information content (PIC = 0.3539) (Table S5 in S1 File). These population genetic parameters confirmed that the selected SSR markers efficiently captured inherent genetic variation within the examined soybean germplasm panel. As powerful molecular genotyping markers, SSR markers have been widely implemented to characterize genetic diversity and evaluate germplasm resources in soybean [28]. In a comparable previous investigation employing 14 SSR primer pairs, the authors reported average PIC and H values of 0.3357 and 0.4121, respectively [29]. In the present study, mean PIC and H were comparable to these published estimates, further validating the credibility of our marker screening and genotyping dataset [30]. Collectively, abundant genetic diversity harbored in the studied soybean accessions lays a fundamental genetic foundation for future high-yield-oriented breeding programmes.

Analyses in this study revealed discrepancies between clustering patterns based on simple sequence repeat (SSR) molecular markers and those derived from phenotypic data (Figs 1 and 3). In line with well-established principles in plant population genetics, such discordance between neutral marker-based and phenotype-based clustering represents a widely documented biological pattern rather than an anomalous outcome. As neutral molecular markers, SSRs primarily capture genome-wide signals of neutral evolutionary processes, including genetic drift and demographic history [31]. In contrast, the agronomic traits assayed in this study are quantitative adaptive phenotypes whose expression is jointly shaped by intrinsic genetic architecture and environmental regulation. Consistent with the results of SSR-based clustering and principal coordinate analysis (PCoA), population structure analysis partitioned the entire germplasm panel into three distinct genetic groups, with the vast majority of accessions assigned to a single dominant subgroup, indicative of a shared core genetic background across the assayed germplasm. Quantification of membership coefficients indicated that 16 accessions were classified as admixed genotypes, reflecting pervasive genetic introgression among the tested materials. The observed discordance between molecular and phenotypic clustering can be largely attributed to the pronounced genotype-by-environment (G×E) interaction detected in this study. Although the two experimental sites share broadly comparable agronomic conditions, subtle differences in field management practices may contribute to divergent phenotypic performance of plants, which represents an important driver underlying the marked differences in the sets of significant association loci detected by the GLM and MLM models across the two locations. This environment-specific genetic regulation of adaptive traits alters the relative phenotypic performance of individual accessions across environments, thereby reshaping phenotypic clustering patterns and decoupling them from neutral genetic groupings. Even minor environmental perturbations can modulate the expression of quantitative traits, consequently altering the outcomes of phenotype-based clustering [32,33]. Furthermore, no significant association was detected between molecular clustering patterns and the geographical origin of the accessions, a finding consistent with previous reports in soybean [34] and other crop species [35,36]. This pattern is driven by two primary categories of factors: evolutionary factors and anthropogenic factors. First, genetic drift over long evolutionary timescales has resulted in overlapping gene pools among geographically isolated germplasm populations [37]. Second, extensive cross-regional germplasm exchange, artificial selection, natural outcrossing, and mechanical seed admixture have further weakened the association between population genetic structure and geographic provenance [38].

Genetic-distance-based UPGMA clustering and STRUCTURE analysis are two independent clustering methods for experimental accessions. UPGMA clusters germplasm on the basis of pairwise genetic distance or genetic similarity and is susceptible to human-derived errors mainly arising from manual genotype identification, whereas STRUCTURE allocates individual materials with ambiguous genetic backgrounds into corresponding genetic populations via molecular genotypic data [39,40]. Combining these two clustering methods facilitates more accurate determination of genetic relationships among germplasm resources [41]. UPGMA clustering grouped 150 germplasm resources into three major categories; while this result captures genetic associations, it may be influenced by operational choices. STRUCTURE software, based on the maximum likelihood principle, identified three clusters (K = 3) corresponding to a peak ΔK value, thereby validating the clustering outcome derived from UPGMA analysis (Fig 5). All three distinct groups identified display genetic admixture within each cluster. It is inferred that the mixed-genotype accessions with complex genetic backgrounds are likely hybrid progeny of other groups or have experienced gene flow, indicating substantial genetic divergence among accessions within the association population. The presence of mixed genotypes in the tested materials results in a relatively simple natural population structure, which effectively mitigates the impact of population structure on association analysis [42].

Association analysis methods based on the general linear model (GLM) and mixed linear model (MLM) have demonstrated significant advantages in multiple crop species [43–45]. In the association analysis of this study, 20 marker loci were repeatedly detected as significantly associated with 12 key yield-related traits under both the GLM and MLM in environment E1, explaining 2.47%−5.66% of the phenotypic variation. Among these loci, seven were simultaneously associated with two or more traits. In environment E2, repeated detection under the same two models (GLM and MLM) identified another 20 marker loci significantly associated with the 12 key yield-related traits, which explained 2.44%−6.30% of the phenotypic variation (S1-12 Figs in S2 File; S7-S18 Tables in S1 File). Although the phenotypic variance explained (PVE) by individual SSR markers was relatively low (2%−6%), which is typical for quantitative traits controlled by multiple minor‑effect loci, these markers can still be used in combination for genomic selection and germplasm evaluation, despite their limited utility for single‑marker‑assisted selection (MAS). Six of these loci showed simultaneous association with two traits. These results indicate close genetic correlations between different agronomic traits and indirectly support the occurrence of pleiotropy [46,47]. Traditional crop breeding primarily relies on phenotypic trait-based selection, which typically results in long breeding cycles and low efficiency [48]. In contrast, identifying allelic loci tightly linked to target traits using molecular markers can significantly improve breeding efficiency and accelerate the breeding process [49]. Notably, six of the associated marker loci were repeatedly detected across the two growing environments (E1 and E2) using both the GLM and MLM. Specifically, Satt592 was significantly associated with pod width; Satt220 and Satt278 were significantly associated with grains per plant; Satt302 was significantly associated with seed weight per plant; Satt005 and Satt316 were significantly associated with hundred-seed weight. Among these, the marker Satt316, which was identified to be associated with hundred-seed weight, has also been reported in previous studies [50]. This study employed 20 evenly distributed, highly polymorphic SSR markers to assess 150 soybean accessions. Despite the relatively low marker density and small phenotypic contribution of the detected markers, which may limit resolution for genome‑wide association analysis, SSR markers remain reliable for preliminary evaluation of genetic diversity and population structure, supporting the main conclusions. Since phenotypic data were collected only in the 2024 growing season, the stability of yield‑related traits across years could not be evaluated, and single‑year data may limit the robustness of the results. Future studies will adopt higher‑density genotyping and multi‑year, multi‑location trials to improve reliability and resolution.

## Conclusion

This study employed phenotypic agronomic traits and SSR markers to characterize genetic diversity, population structure, and marker-trait associations across 150 Chinese soybean germplasm accessions, providing a theoretical reference for germplasm authentication, innovative utilization and molecular breeding. Phenotypic evaluation uncovered abundant phenotypic variation within the tested panel, implying promising breeding potential for yield improvement. Collectively, our phenotypic and molecular assessments comprehensively clarified the genetic variability of these germplasm resources and accomplished the core research objective of diversity evaluation. Combining phenotypic phenotyping with genome-wide SSR-based association analysis, GLM and MLM models consistently detected significant SSR markers linked to four yield-related traits including pod width, seed number per plant, total seed weight per plant and 100-seed weight. Nevertheless, further fine-mapping of relevant candidate genomic intervals is necessary to excavate yield-improving genes for subsequent soybean high-yield breeding and follow-up functional research.

## Supporting information

S1 File S1-S18 Table. Contain comprehensive experimental data in sequence, including the origins of test materials, SSR primer information, genetic diversity and cluster mean values of agronomic traits, SSR primer polymorphism, population structure Q-values, as well as the full association analysis results between ten soybean agronomic traits and SSR markers.
(XLSX)

S2 File S1-S12 Fig. SSR molecular marker loci associated with 12 agronomic traits.
(DOCX)

S3 File S13-24 Fig. Q-Q plot of SSR marker association analysis for12 agronomic traits.
(DOCX)

S4 Fig S25 Fig. Raw image of Figure 2.
(PDF)
