# Supplementary material for: Genetic diversity and yield trait associations in soybean germplasm using SSR markers
Source: PLoS One. 2026 Aug 4;21(8):e0355194. doi: 10.1371/journal.pone.0355194 (PMC13436762; doi:10.1371/journal.pone.0355194)
Supplement: Supplementary file 3 [file pone.0355194.s002.docx]

Supplementary Material

# 1 Supplementary Figures

**S1 Fig.** SSR molecular marker loci associated with plant height. (a) and (b) show the Manhattan plots of SSR marker loci significantly associated in E1 and E2, respectively, using the GLM model (p<0.05). (b) and (c) show the Manhattan plots of SSR marker loci significantly associated in E1 and E2, respectively, using the MLM model (p<0.05). The black dashed line indicates the screening threshold for significantly associated SSR marker sites (p=10⁻¹³). The x-axis (A-T) denotes the SSR molecular marker primer numbers; the same applies below.

**S2 Fig.** SSR molecular marker loci associated with pod heights.

**S3 Fig.** SSR molecular marker loci associated with number of main stem nodes.

**S4 Fig.** SSR molecular marker loci associated with pods per plant.

**S5 Fig.** SSR molecular marker loci associated with stem diameter.

**S6 Fig.** SSR molecular marker loci associated with pod length.

**S7 Fig.** SSR molecular marker loci associated with pod width.

**S8 Fig.** SSR molecular marker loci associated with seeds per plant.

**S9 Fig.** SSR molecular marker loci associated with seed weight per plant.

**S10 Fig.** SSR molecular marker loci associated with hundred-seed weight.

**S11 Fig.** SSR molecular marker loci associated with seed length.

**S12 Fig.** SSR molecular marker loci associated with seed width.
