# Supplementary material for: Genetic diversity and yield trait associations in soybean germplasm using SSR markers
Source: PLoS One. 2026 Aug 4;21(8):e0355194. doi: 10.1371/journal.pone.0355194 (PMC13436762; doi:10.1371/journal.pone.0355194)
Supplement: Supplementary file 4 [file pone.0355194.s003.docx]

Supplementary Material

# 1 Supplementary Figures

**S13 Fig.** Q-Q plot of SSR marker association analysis for plant height.

(a) and (b) show the Q-Q plots for agronomic traits in E1 and E2, respectively, following GLM and MLM model analyses of the SSR marker loci. The same applies below.

**S14 Fig.** Q-Q plot of SSR marker association analysis for pod height.

**S15 Fig.** Q-Q plot of SSR marker association analysis for main stem nodes.

**S16 Fig.** Q-Q plot of SSR marker association analysis for pod per plant.

**S17 Fig.** Q-Q plot of SSR marker association analysis for stem diameter.

**S18 Fig.** Q-Q plot of SSR marker association analysis for pod length.

**S19 Fig.** Q-Q plot of SSR marker association analysis for pod width.

**S20 Fig.** Q-Q plot of SSR marker association analysis for seeds per plant.

**S21 Fig.** Q-Q plot of SSR marker association analysis for seed weight per plant.

**S22 Fig.** Q-Q plot of SSR marker association analysis for hundred-seed weight.

**S23 Fig.** Q-Q plot of SSR marker association analysis for seed length.

**S24 Fig.** Q-Q plot of SSR marker association analysis for seed width.
