# Supplementary material for: Genetic diversity and yield trait associations in soybean germplasm using SSR markers
Source: PLoS One. 2026 Aug 4;21(8):e0355194. doi: 10.1371/journal.pone.0355194 (PMC13436762; doi:10.1371/journal.pone.0355194)
Supplement: Supplementary file 5 [file pone.0355194.s005.docx]

**Reviewer report**

The manuscript presents a solid analysis of genetic diversity and SSR-based marker-trait associations in soybean germplasm evaluated across two environments. The study is technically sound and the results are clearly presented. However, the novelty is limited, and several methodological issues require clarification and discussion. Addressing these points would improve the rigor and interpretability of the findings.

**Comments**

1. While this manuscript does a solid job using SSR markers to map soybean genetic diversity, it’s currently missing a clear 'hook.' Since high-density SNP data is now the standard, the authors really need to highlight what makes this specific study unique. What does this research tell us that previous SSR studies missed? Strengthening the Introduction to clearly state this novelty would make the paper much more compelling (Lines 11-31).

2. Since the phenotypic data comes from just the 2024 season, it’s difficult to tell how much these yield traits fluctuate due to the environment. Because yield is so sensitive to outside factors, the results would be much more robust if we could see trait stability over time. At the very least, the authors should be upfront about this limitation in the discussion and explain how it might influence their conclusions (Lines 87-96).

3. The two test sites are geographically close and share similar weather patterns, I’m wondering if they actually provided enough environmental contrast for the study. It would be helpful if the authors could clarify how distinct these sites really are - otherwise, it becomes difficult to accurately interpret the genotype × environment interactions. A bit more detail here would help justify the experimental design. (Lines 87-92).

4. DNA was extracted from pooled leaf samples per accession. While this approach reduces intra-accession variation, it may also mask within-accession heterogeneity. The rationale and potential consequences of this strategy should be discussed. (Lines 97-100).

5. Using 20 SSR markers to characterize 150 accessions provides a relatively thin look at the genome. With such low marker density, there’s a risk that we’re missing a lot of the genetic landscape, which could make the diversity estimates and association results less reliable. The authors should explicitly address how this limited coverage might impact the strength of their conclusions. (Lines 123-129).

6. The transition from the STRUCTURE analysis to the TASSEL MLM needs more detail. Specifically, the authors should explain how they incorporated the Q and K matrices into their model. Providing the exact configuration used will help clarify how they controlled for population stratification and relatedness. (Lines 161-168; 271-273).

7. The authors set the significance threshold at *P* < 0.05, but they didn't account for multiple testing. When you're looking at several traits across multiple markers, the odds of finding 'significant' hits by pure chance go way up. To make these associations more believable, the authors should either justify why a raw *p*-value is sufficient here or, better yet, apply a correction like FDR or Bonferroni to filter out potential false positives. (Lines 170-172).

8. Although the analyses suggest a possible relationship between genetic clustering and geographic origin, this aspect is not explored in depth. The authors are encouraged to examine this relationship more explicitly, for example by assessing whether geographic origin meaningfully contributes to the observed population structure or whether extensive admixture weakens this association. Clarifying this point would substantially strengthen the population genetic interpretation. (Lines 231-238).

9. The UPGMA, PCoA, and STRUCTURE analyses reveal largely consistent patterns. To improve readability, the Results section could be streamlined by reducing repetitive descriptions and instead highlighting the complementary insights provided by each analytical approach. (Lines 231-268).

10. To get a better sense of how well the PCoA represents the actual data, it would be really helpful if the authors could include the percentage of variance explained by the first three principal coordinates. Without those numbers, it’s hard for the reader to tell how much of the genetic story is actually being captured by the plot and how much is just 'noise. (Lines 243-251).

11. Most associated markers account for only a small proportion of the phenotypic variation (around 2–6%). A more critical discussion of their biological significance and practical value for breeding would strengthen the interpretation of these results. (Lines 287-296).

12. Several sections of the Discussion repeat similar points about genetic diversity and the effectiveness of SSR markers. Condensing these passages would improve clarity and allow greater focus on interpretation rather than repetition. (Lines 298-358).

13. The authors report moderate values for PIC, gene diversity, and effective allele number, which is a good start. However, to really understand what these numbers mean for this specific population, it would be helpful to see them compared to previous soybean diversity studies. Placing these results in the context of earlier SSR-based research would clarify whether this germplasm is more or less diverse than what's typically seen in the field. (Lines 341-345).

14. The association results would be strengthened by a clearer comparison with previously published soybean GWAS or QTL studies on yield-related traits. (Lines 391-409).

15. While the manuscript reports stable SSR–trait associations, these markers are not linked to known genomic regions or candidate genes. Even a brief discussion placing them in a genomic context would strengthen the study. (Lines 403-420).

16. Terms such as “hundred-grain weight” and “hundred-seed weight” are used interchangeably. Please standardize terminology throughout. (Throughout manuscript).

17. The manuscript is generally well written, but some sentences – especially in the Discussion – are overly long and repetitive. Careful language editing would improve readability.
