# Supplementary material for: Genetic diversity and yield trait associations in soybean germplasm using SSR markers
Source: PLoS One. 2026 Aug 4;21(8):e0355194. doi: 10.1371/journal.pone.0355194 (PMC13436762; doi:10.1371/journal.pone.0355194)
Supplement: Supplementary file 6 [file pone.0355194.s007.docx]

**Response Letter**

Manuscript ID: 1710481

Dear Editor and Reviewers:

We appreciate the constructive feedback provided by the editor and reviewers on our manuscript, which has played a crucial role in improving the quality of our work. We have carefully considered all comments and made the necessary revisions on the cover letter and the manuscript. The following provides detailed response to each of the comments.

**Reviewer #1:**

The manuscript presents a solid analysis of genetic diversity and SSR-based marker-trait associations in soybean germplasm evaluated across two environments. The study is technically sound and the results are clearly presented. However, the novelty is limited, and several methodological issues require clarification and discussion. Addressing these points would improve the rigor and interpretability of the findings.

Comments

**1. While this manuscript does a solid job using SSR markers to map soybean genetic diversity, it’s currently missing a clear 'hook.' Since high-density SNP data is now the standard, the authors really need to highlight what makes this specific study unique. What does this research tell us that previous SSR studies missed? Strengthening the Introduction to clearly state this novelty would make the paper much more compelling (Lines 11-31).**

**Response:** We thank the reviewer for the constructive suggestion. We have revised the Abstract (Lines 11-31) to clearly highlight the novelty and unique value of this study. Specifically, we have clarified that while high-density SNP data is now the standard, our SSR-based analysis has its distinct advantages and fills a gap in previous SSR-related studies on soybean genetic diversity. We have also shortened overly long sentences and removed repetitive content in the Abstract to further improve readability and compellingness.

**2. Since the phenotypic data comes from just the 2024 season, it’s difficult to tell how much these yield traits fluctuate due to the environment. Because yield is so sensitive to outside factors, the results would be much more robust if we could see trait stability over time. At the very least, the authors should be upfront about this limitation in the discussion and explain how it might influence their conclusions (Lines 87-96).**

**Response:** We thank the reviewer for this valuable comment. We have added a clear statement in the Discussion to acknowledge the limitation of single-year phenotypic data and its potential impact on the conclusions. We have also indicated that multi-year trials will be needed to improve the robustness of the results in future research.

**3. The two test sites are geographically close and share similar weather patterns, I’m wondering if they actually provided enough environmental contrast for the study. It would be helpful if the authors could clarify how distinct these sites really are-otherwise, it becomes difficult to accurately interpret the genotype×environment interactions. A bit more detail here would help justify the experimental design. (Lines 87-92).**

**Response:** We thank the reviewer for this valuable comment. We have clarified in the manuscript that the two test sites are geographically close with similar climatic conditions, and we have noted the potential influence on the interpretation of genotype×environment interactions. We have also added a justification and outlook for further experiments with more contrasting environments.

**4. DNA was extracted from pooled leaf samples per accession. While this approach reduces intra-accession variation, it may also mask within-accession heterogeneity. The rationale and potential consequences of this strategy should be discussed. (Lines 97-100).**

**Response:** We appreciate the reviewer’s thoughtful comment. In this study, we used bulked leaf tissue per accession for DNA extraction to minimize the impact of random intra-accession variation and ensure representative genotyping for each accession, which is a common and widely accepted practice in genetic diversity and association analyses using germplasm collections. Although this method may mask minor within-accession heterogeneity, it does not affect the core conclusions of genetic diversity, population structure, and marker-trait association analysis at the accession level, which were the primary focuses of this study.

**5. Using 20 SSR markers to characterize 150 accessions provides a relatively thin look at the genome. With such low marker density, there’s a risk that we’re missing a lot of the genetic landscape, which could make the diversity estimates and association results less reliable. The authors should explicitly address how this limited coverage might impact the strength of their conclusions. (Lines 123-129).**

**Response:** We appreciate the reviewer’s thoughtful comment. In this study, we selected 20 highly polymorphic SSR markers. Although marker density is lower than that of high-density SNP platforms, SSR markers show high reproducibility and codominant inheritance. They have sufficient discriminatory power for genetic diversity evaluation and are widely accepted in published studies. Limited genome coverage may reduce the resolution of association analysis, but it does not affect the estimation of genetic diversity, population structure, or preliminary screening of associated markers, which are the main objectives of this study. We have added this discussion in the revised manuscript.

**6. The transition from the STRUCTURE analysis to the TASSEL MLM needs more detail. Specifically, the authors should explain how they incorporated the Q and K matrices into their model. Providing the exact configuration used will help clarify how they controlled for population stratification and relatedness. (Lines 161-168; 271-273).**

**Response:** We appreciate the reviewer’s constructive comments. We have revised the description of association analysis models. The content has been simplified, and the model formulas have been replaced with clear textual descriptions to improve readability and conciseness.

**7. The authors set the significance threshold at *P*<0.05, but they didn't account for multiple testing. When you're looking at several traits across multiple markers, the odds of finding 'significant' hits by pure chance go way up. To make these associations more believable, the authors should either justify why a raw p-value is sufficient here or, better yet, apply a correction like FDR or Bonferroni to filter out potential false positives. (Lines 170-172).**

**Response:** We greatly appreciate the reviewer’s valuable comment on multiple testing correction, which is critical for ensuring the reliability of association analysis. We fully acknowledge that uncorrected P-values may increase the risk of false positives.

However, given the specific objectives and marker density of this study, we retained the nominal significance threshold of P < 0.05 for the following reasons: Only 20 SSR markers were used in this study, which is far fewer than typical genome-wide marker sets; This study aimed to conduct an exploratory association analysis to identify preliminary candidate markers rather than to draw definitive conclusions; Overly strict correction would lead to an extremely high threshold and greatly increase the risk of false negatives, which would mask potentially meaningful associations.

**8. Although the analyses suggest a possible relationship between genetic clustering and geographic origin, this aspect is not explored in depth. The authors are encouraged to examine this relationship more explicitly, for example by assessing whether geographic origin meaningfully contributes to the observed population structure or whether extensive admixture weakens this association. Clarifying this point would substantially strengthen the population genetic interpretation. (Lines 231-238).**

**Response:** We thank the reviewer for this constructive suggestion. We have further discussed the relationship between genetic clustering and geographic origin in the Discussion. We have clarified the contribution of geographic origin to population structure and the influence of genetic admixture, which strengthens the population genetic interpretation of this study.

**9. The UPGMA, PCoA, and STRUCTURE analyses reveal largely consistent patterns. To improve readability, the Results section could be streamlined by reducing repetitive descriptions and instead highlighting the complementary insights provided by each analytical approach. (Lines 231-268).**

**Response:** We appreciate the reviewer’s suggestion to streamline the Results section. We have revised the descriptions of UPGMA, PCoA, and STRUCTURE analyses by removing redundant and repetitive content, while keeping the three methods presented separately for clarity. Each method now highlights its unique and complementary role in inferring population structure, making the results more concise and readable.

**10. To get a better sense of how well the PCoA represents the actual data, it would be really helpful if the authors could include the percentage of variance explained by the first three principal coordinates. Without those numbers, it’s hard for the reader to tell how much of the genetic story is actually being captured by the plot and how much is just 'noise. (Lines 243-251).**

**Response:** We appreciate the reviewer’s valuable suggestion.Accordingly, we have added the percentage of variance explained by the first three principal coordinates in the figure legend of PCoA (Fig. 4). This allows readers to better evaluate the quality and explanatory power of the PCoA analysis.
**11. Most associated markers account for only a small proportion of the phenotypic variation (around 2–6%). A more critical discussion of their biological significance and practical value for breeding would strengthen the interpretation of these results. (Lines 287-296).**
**Response:** We thank the reviewer for this insightful comment.We have added a more in-depth discussion regarding the biological meaning and breeding relevance of the associated markers, especially addressing their relatively small phenotypic variance explained. This additional discussion improves the interpretation of our association results.

**12. Several sections of the Discussion repeat similar points about genetic diversity and the effectiveness of SSR markers. Condensing these passages would improve clarity and allow greater focus on interpretation rather than repetition. (Lines 298-358).**
**Response:** Thank you for pointing out the repetitive content in the Discussion. We have carefully condensed the sections discussing genetic diversity and SSR marker effectiveness, removing all redundant descriptions. The revised manuscript now focuses more on the interpretation of our findings rather than repetitive summaries, which has enhanced the overall clarity and readability of this section.

**13. The authors report moderate values for PIC, gene diversity, and effective allele number, which is a good start. However, to really understand what these numbers mean for this specific population, it would be helpful to see them compared to previous soybean diversity studies. Placing these results in the context of earlier SSR-based research would clarify whether this germplasm is more or less diverse than what's typically seen in the field. (Lines 341-345).**

**Response:** We thank the reviewer for the helpful comment. We have added comparisons between our PIC, gene diversity, and effective allele number values and those reported in previous soybean SSR studies. This context clarifies the level of genetic diversity in our germplasm relative to previously reported populations.

**14. The association results would be strengthened by a clearer comparison with previously published soybean GWAS or QTL studies on yield-related traits. (Lines 391-409).**

**Response:** We appreciate the reviewer’s valuable suggestion regarding the comparison of our association results with previous GWAS and QTL studies. In this study, we used a relatively small number of SSR markers distributed across the genome, rather than a high‑density genotyping platform typically used in GWAS. Because of the limited marker density and coverage, our results are more suitable for preliminary marker‑trait association detection instead of direct comparison with high‑resolution GWAS or QTL results. We have clarified this point in the revised discussion to better highlight the purpose and scope of the present study.

**15. While the manuscript reports stable SSR–trait associations, these markers are not linked to known genomic regions or candidate genes. Even a brief discussion placing them in a genomic context would strengthen the study. (Lines 403-420).**

**Response:** We appreciate the reviewer’s constructive comment. In this study, we used a limited set of SSR markers for preliminary association analysis, rather than high-density genotyping platforms employed in conventional GWAS. Due to the low marker density and insufficient genome coverage, we could not accurately anchor the associated signals to specific genomic regions or known candidate genes. We have clarified this limitation and emphasized the preliminary nature of these associated markers in the revised discussion. More precise mapping and gene identification will be carried out in future studies using high-density markers and larger populations.

**16. Terms such as “hundred-grain weight” and “hundred-seed weight” are used interchangeably. Please standardize terminology throughout. (Throughout manuscript).**

**Response:** We thank the reviewer for this reminder. We have standardized the trait name 100-seed weight throughout the entire manuscript and removed inconsistent expressions such as hundred-grain weight.

**17. The manuscript is generally well written, but some sentences-especially in the Discussion-are overly long and repetitive. Careful language editing would improve readability.**

**Response:** We appreciate the reviewer’s positive comments and constructive suggestion. We have carefully revised the manuscript, especially the Discussion section, by shortening overly long sentences and removing repetitive content. Language editing has been performed throughout to improve clarity and readability.

**Reviewer #2:**

The authors used SSR markers to study genetic diversity and yield trait correlations in soybean germplasm. The study contains no new information. The manuscript needs multiple modifications.

**Abstract**

**• The issue of the study is unavailable.**

**• Some scored data about the morphological traits should be included**

**• The abbreviated SSR should be defined in full name**

**• A brief conclusion should be written at the end of the abstract**

**Response:**

We thank the reviewer for the constructive comments. We have added a clear research issue in the manuscript, supplemented statistical data of morphological traits, defined the full name of SSR at first use, and added a brief conclusion at the end of the abstract.

**Keywords**

**The words used for the creation of the title should not be used as the keywords**

**Response:**

We have revised the keywords to avoid repetition with the title, and selected more representative and standardized terms.

**Introduction**

**• Recent references should be used**

**• The authors should provide some lines about genetic diversity methods.**

**• The methods of association between markers and quantitative traits should be stated**

**• The gap in the research should be highlighted.**

**• The hypothesis of the study should be determined**

**• Originality of the work should be added**

**Response:**

We appreciate the reviewer’s constructive suggestions. We have further optimized the introduction section by supplementing recent relevant references, briefly introducing common methods for genetic diversity detection and marker-trait association analysis of quantitative traits, and emphasizing the existing research gaps. We have also clearly stated the research hypothesis and highlighted the novelty and practical significance of this work.

**Materials and Methods**

**• All methods used should be supported by the references.**

**• The manufacture of all materials and instruments should be defined**

**• All abbreviations should be written in full**

**Response:**

We appreciate the reviewer’s comments. Relevant references have been supplemented to support the adopted methods. The sources and manufacturers of key instruments and reagents have been clearly stated. All abbreviations have been checked and provided with their full names at first use in the manuscript.

**Results and Discussion**

**• The significant status of each measurement should be stated**

**• The details of the results in the tables and figures must be documented.**

**• All captions should be improved**

**• The genetic diversity indices should be performed for populations.**

**• The gene flow and fixation index should be computed**

**• The discussion is poor and should be improved. There are a few repeated lines. The contributors should analyze the link between all researched features by explaining the minimum and maximum results. The writers should interpret the relationship between each of the parameters being studied.**

**Response:**

We sincerely appreciate the reviewer’s valuable suggestions. We have clearly stated the significance levels of all measured indicators in the manuscript. The detailed information of all tables and figures has been supplemented, and all captions have been improved and standardized. In addition, we have comprehensively revised the discussion section, deleted repeated content, and deeply analyzed the maximum and minimum values of traits, as well as the relationships among all tested parameters.

**Conclusions**

**• Conclusions should include the most important findings. Additional works should be included.
Response:**

We have rewritten the conclusion section to highlight the key findings and provided suggestions for future research.

**Reviewer #3:**

The study aims to evaluate the genetic diversity, and marker trait associations between SSR markers and yield related traits in 150 soybean accessions. The study utilized 20 pairs of SSR primers to genotype diverse soybean accessions. The study was conducted across two environmental conditions.

**This study has following strengths:**

**1. The objective “understanding genetic diversity and identifying marker–trait associations in soybean” is clearly stated and highly relevant to breeding programs.**

**2. The authors specify the number of SSR primers (20), sample size (150 accessions), and the use of two environmental conditions.**

**3. The inclusion of multiple analytical methods (GLM, MLM, cluster analysis, PCoA, population structure analysis) shows methodological rigor.**

**4. The study provides detailed quantitative results (e.g., allele numbers, PIC values, polymorphism rates). Identifying 44 markers associated with yield traits and 6 consistently across environments is a strong highlight.**

**5. The intended application is clearly stated highlighting the practical value of the study.**

**Response:**

We greatly appreciate the reviewer for recognizing the strengths, scientific value, and practical significance of this study. We have carefully revised the manuscript according to the constructive comments to further improve its quality.

Following are few suggestions:

**1. Twelve key agronomic traits are mentioned in the abstract. Do add some specific details (what they are? Some description related to substantial phenotypic variation?).**

**Response:**

We have supplemented the specific composition of the 12 key agronomic traits and added detailed descriptions of their abundant phenotypic variation in the abstract.

**2. Line 30: What you mean by “future conservation management” and “scientific breeding”? Can you replace these with some more familiar terms?**

**Response:**

We greatly appreciate the reviewer’s suggestion. To make the expression clearer and more understandable, we have revised the terms

**3. Line 34: Provide reference related to “biofuel production”.**

**Response:**

We greatly appreciate the reviewer’s suggestion. To make more understandable, We have made the modifications.

**4. Line 45 to 47: Simplify the statement “Substantial beneficial ……………..”**

**Response:**

We greatly appreciate the reviewer’s suggestion. To make more understandable, we We have made the modifications.

**5. Line 48 and 51: Is using the term “divergent” or “diverse” right here?**

**Response:**

We thank the reviewer for this careful comment. To ensure clarity and accuracy, we have revised and standardized the wording throughout the manuscript to avoid ambiguity.

**6. Line 64: Please provide reference.**

**Response:**

We thank the reviewer for the careful and detailed corrections. The issue has been revised.

**7. Line 68: Please provide reference.**

**Response:**

We thank the reviewer for the careful and detailed corrections. The issue has been revised.

**8. Line 69: Please use some simple word instead of “corroborated”**

**Response:**

We appreciate the reviewer’s suggestion and made modifications.

**9. Line 75: Use some other word instead of “safeguarding”.**

**Response:**

We appreciate the reviewer’s suggestion and made modifications.

**10. Line 88 and 90: Please explain why these environments (E1 and E2) were chosen for this study? How these environments are different? Based on environmental factors, do they differ? Instead of using two similar types of environments, wasn’t it better to go for multi-year study?**

**Response:**

Thank you for your opinion. We describe the main differences between the two environments in the Materials and Methods section. Although both sites have sandy loam soil, they differ in **latitude, longitude, and key climatic factors** during the 2024 growing season.

E1 (Hailiutu Science and Technology Park, 40°68′N, 111°37′E) had a mean temperature of 18.5℃, precipitation of 420 mm, and relative humidity of 60%. E2 (East Science and Technology Park, 40°81′N, 111°72′E) had a mean temperature of 17.8℃, precipitation of 390 mm, and relative humidity of 58%. These differences provided sufficient environmental variation for evaluating phenotypic performance and genotype–environment interaction in the present study.

Regarding the multi‑year study suggestion:

We fully agree that multi‑year, multi‑location experiments can further improve the reliability and universality of results. In this study, we focused on genetic diversity, population structure, and marker‑trait association analysis using one‑year phenotypic data from two contrasting sites. In future work, we will conduct multi‑year and multi‑location trials to validate the stability of our findings and strengthen their application in soybean breeding.

**11. In results and discussion, please emphasize on interpretations. For example, what do the high polymorphism rates imply? How does clustering across environments support your conclusions? A sentence tying the results to broader implications would strengthen the narrative.**

**Response:**

We greatly appreciate the reviewer’s constructive suggestion and have incorporated them into our discussion.

**Conclusion/Recommendations**

**1. Overall, the study presents promising findings. The article is concise and well-written.**

**2. Recommendation: Minor Revision**

**Response:**

Thank you for your review.

Reviewer #4:

The research article entitled “Genetic Diversity and Yield Trait Associations in Soybean Germplasm Using SSR Markers” is generally well organized, and the research objectives, methodology, and results are presented in a logical sequence. Most sections are understandable, and the figures and tables appropriately support the text. The manuscript has presented valuable data on Chinese soybean germplasm. However, following comments should be addressed to improve the scientific rigor and clarity of the study.

Major Comments:

**1. The field study is only for one year with two locations. Multi-year or multi-location data would strengthen study, and authors should acknowledge this limitation.**

**Response:** We agree with the reviewer that multi-year and multi-location experiments can improve the reliability and universality of the results. In the revised manuscript, we have added a statement acknowledging this limitation in the Discussion section. We clarify that the phenotypic data were collected from two locations in a single year, and that multi-year trials will be conducted in future studies to further validate the stability of the associated markers and phenotypic performance.

**2. As the study is comprehensive, however there is need to highlight novel markers associations and the special germplasm. The authors should emphasize how their findings extend beyond previously published soybean SSR studies.**

**Response:**

In the revised Discussion, we have strengthened the description of the novel associated markers and elite germplasm resources identified in this study.

**3. PVE values for SSR markers are relatively low (2–6%), authors should discuss the implications for breeding and whether these loci are sufficiently informative for MAS. For instance, in table S6, PVE ranges from 3.22% to 3.90% for the trait plant height indicating that each SSR marker explains only a small fraction of variation in plant height. While this is expected for complex quantitative traits, the manuscript does not adequately discuss the limited practical utility of such low-effect markers for marker-assisted selection. This limitation should be explicitly acknowledged.**

**Response:** We appreciate the reviewer’s professional and valuable comment. The PVE values of the identified SSR markers ranged from 2% to 6%, which were relatively low. As noted by the reviewer, for complex quantitative traits such as plant height, individual markers often explain only a small proportion of phenotypic variation. In the revised manuscript, we have added a detailed discussion addressing the implications of low PVE values for breeding applications and explicitly acknowledged that markers with small individual effects have limited direct utility for single-marker marker-assisted selection (MAS). We also clarified that these loci can still be informative when used in combination for genomic selection or germplasm characterization. This limitation has been clearly stated in the Discussion section.

**4. The supplementary tables report nominal P-values (<0.05), but there is no indication that multiple testing correction (e.g., Bonferroni or FDR) was applied. Given the large number of markers–trait tests typically performed, the absence of correction raises concerns about inflated Type I error.**

**Response:** We appreciate the reviewer’s concern regarding multiple testing correction. In the present study, we used a nominal threshold of *P*<0.05 to identify preliminary marker–trait associations, as is commonly adopted in SSR-based association mapping studies with a limited number of markers, rather than a genome-wide panel.

**5. Several markers identified by GLM (are not confirmed by MLM, suggesting potential false positives due to population structure effects. Authors should be cautious in reporting such association and they should emphasize MLM-validated markers. e.g., Satt302 and Satt278 Trait: Pods per Plant**

**Response:** We appreciate the reviewer’s concern about false positives caused by population structure. In this study, both GLM and MLM models were employed for comparative analysis of marker-trait associations. Although some markers (such as Satt302 and Satt278 for pods per plant) were detected only by GLM but not by MLM, we emphasize that GLM remains a commonly used model for initial screening in SSR-based association studies, particularly for exploring potential loci that might be overlooked in MLM due to overcorrection of population structure. These GLM-specific associations are not interpreted as definitive or stable QTL, but are regarded as preliminary candidate loci for further validation. The manuscript also highlights numerous sites detected by both GLM and MLM models.

**6. Needs to recheck Table S1 critically about the total germplasm, 150 0r 148, as some lines such as NF867 and HH705 are repeated. If the accessions are repeated, then write the paper accordingly.**

**Response:** We sincerely apologize for the error in Supplementary Table S1. After meticulous verification of all germplasm resources, we confirm that duplicate entries (NF867 and HH705) were indeed present in the original table. The revised version has corrected these duplicate accessions. Consequently, the total number of soybean germplasm resources used in this paper has been amended to 150 accessions. We extend our heartfelt gratitude to the reviewers for their meticulous identification of this error, which has significantly contributed to enhancing the accuracy and reliability of our manuscript.

Minor Comments:

**• Need to add more columns in Table S1 such as “Type” (e.g., landrace, improved variety), accession numbers, collection years, or source institutions. Such information is often expected in germplasm tables to allow reproducibility and traceability.**

**• Some germplasm names include spaces, hyphens, or inconsistent capitalization, which needs uniformity which improves professionalism.**

**• Table S2: Position” is ambiguous. Does it refer to chromosomal position? Need to clarify.**

**• Table S2: There is a typo in row 5 (Annealing Temperature: 561 ℃), which is clearly impossible. Such errors reduce confidence in the data.**

**• Needs uniformity, “Sat_385” and “Satt220.” why some use underscores, some do not? Need to re-check the completed paper**

**• Table S2: Make sure the formatting is consistent (some have extra spaces, e.g., “58.2 ℃”).**

**• Table S3: Some numeric entries, e.g., Hundred-seed weight in E2 (24.94366197), are reported with excessive decimal places. Needs rounding to 2–3 decimal places in all other tables too**

**• Table S3: Need to mention the unit of some agronomic trait such plant height, cm? stem diameter, cm or mm? Units should be included for all the traits in other tables too such as S4**

**• Table S4: The sample IDs (e.g., 38.00, 30.00) are confusing—are these, averages of specific samples? A clear explanation in a footnote is necessary.**

**• The term “Interpretation rate (%)” should be replaced with “Phenotypic variance explained (PVE in all the relevant tables.**

**• While two environments were used, the discussion could more explicitly address environment-specific vs. stable associations, particularly for markers detected in both E1 and E2.**

**• Decimal precision is inconsistent in all the supplementary tables (e.g., P values reported with varying numbers of digits). For instance, in S15, (some have many decimals, e.g., 0.048112905) which is unnecessary; 3–4 decimal places are sufficient.**

**• Need to write either 100-seed weight or hundred-grain weight**

**• Needs to highlight markers detected across both environments and models (GLM and MLM) in all the relevant tables**

**Response:**

We thank the reviewer for the careful and detailed corrections. All minor issues have been revised.
