# Supplementary material for: Genetic diversity and yield trait associations in soybean germplasm using SSR markers
Source: PLoS One. 2026 Aug 4;21(8):e0355194. doi: 10.1371/journal.pone.0355194 (PMC13436762; doi:10.1371/journal.pone.0355194)
Supplement: Supplementary file 7 [file pone.0355194.s009.docx]

**Response Letter**

Manuscript ID: 1710481

Dear Editor and Reviewer:

We appreciate the constructive feedback provided by the editor and reviewer on our manuscript, which has played a crucial role in improving the quality of our work. We have carefully considered all comments and made the necessary revisions on the cover letter and the manuscript. The following provides detailed response to each of the comments.

**Reviewer #5:**

This manuscript presents an interesting study evaluating 150 soybean genotypes using both phenotypic traits and genetic markers across two distinct environmental conditions. The research is highly relevant within the context of crop breeding and aligns well with the economic and agricultural significance of this crop. However, the study also exhibits several flaws, discrepancies, and conceptual inconsistencies that are of fundamental importance to its scientific contribution and overall validity. These critical issues must be thoroughly addressed and resolved before the manuscript can be considered for publication.

Comments

**Line 81–82: In the supplementary material, I noticed that most loci are not stable across the two environments. I highly recommend removing this specific part of the sentence.**

**Response:** We thank the reviewer for this valuable comment. We have removed the relevant sentence regarding the instability of most loci across two environments in the revised manuscript accordingly.

**Lines 102–104: The authors state that they collected 3 leaves per plant. However, it is unclear from how many total plants the leaves were sampled. Were they collected from every replication of the samples and across both environments? If so, the total volume of leaf tissue would be far too large for the commercial extraction kit used. Furthermore, how did this fit into a 5 mL tube, and how can the authors guarantee that taking a tiny 30 mg subsample from such a large bulk mass provides a truly representative sample? There is a lack of clarity here that can be easily resolved.**

**Response:** We greatly appreciate your careful review and constructive suggestions. Corresponding revisions have been made to this section. Given that plant genomes are stable across different environments, we sampled leaves from one single location. After thorough grinding and mixing, random subsampling was performed on the homogeneous material to ensure sample representativeness.

**Line 107: Given that you have three plant replications, please specify how the three random plants were selected. I assume three plants from each environment were used for morphological measurements.**

**Response:** Thank you for the comment. We have added relevant details in the revised manuscript. Consistent with your assumption, three individual plants from each environment were used for morphological trait measurements.

**Line 122: Please clarify how many samples were used and from which specific part of the plant they were taken.**

**Response:** Thanks for pointing out this omission. We have clarified the information in line 102-105 accordingly. Consistent with the above sampling method, a total of 150 samples were adopted, and they were collected from the leaves of individual plants.

**Line 122: Shannon-Weaver diversity index-There is no reference, formula, or software package cited for this method. It is unclear whether a software tool was used or if it was calculated based on a previously described protocol in literature. Please add a formal reference or the exact formula to ensure clarity and reproducibility.**

**Response:** Thank you for this valuable suggestion. We have supplemented the calculation formula of the Shannon-Weaver diversity index in the revised manuscript .

**Line 165: The software version for iTOL is missing.**

**Response:** Thank you for the careful check. We have supplemented the exact software version of iTOL in the revised manuscript. Phylogenetic tree visualization was performed using iTOL v7 (https://itol.embl.de).

**Line 179: Phenotypic variation explained (R2) – This specific abbreviation appears only in the Materials and Methods section, after which it is abruptly changed to "PVE". This inconsistency must be standardized throughout the manuscript.**

**Response:** Thank you for pointing out this inconsistent abbreviation. We have uniformly replaced “phenotypic variation explained (R²)” with “PVE” throughout the full manuscript to unify the terminology.

**Line 142: The text states a fixed annealing temperature of 50°C. However, Supplementary Figure 2 indicates that the annealing temperatures for each primer actually vary from 40.3°C to 60.5°C. Please resolve this discrepancy.**

**Response:** We appreciate the reviewer’s careful inspection. We have revised the description in the manuscript accordingly. Instead of a unified fixed annealing temperature, the annealing temperature of each primer ranged from 40.3 °C to 60.5 °C.

**Figure 2: Figure 2 presents data for only 40 individuals. While I fully understand the technical impossibility of running all samples on a single gel for direct comparison, I strongly recommend adding the remaining gels for at least one marker. If not included as a main figure, this data should be provided as supplementary material. This will allow the reader to gain a clear understanding of how the scoring and sizing of alleles were standardized and compared across separate gels.**

**Response:** Thank you for your valuable feedback. Due to objective limitations of the experimental conditions, the original electrophoresis gel images of the remaining individuals cannot be submitted as supplementary material. This polyacrylamide gel electrophoresis experiment was completed in July 2024. At the time of the experiment, the bands were interpreted manually by visual inspection, and images were captured using early-generation imaging equipment; consequently, complete digital copies of the original images were not retained; At the time of the experiment, gel images from only 40 representative individuals were collected and used for Figure 2.

**Line 265: Supplementary material regarding the Q-values is missing. Since these values are directly utilized in the association analysis, providing this data is essential. Looking at Figure 5, it can be inferred that there are accessions with a Q-value < 0.6, which implies a mixed origin (admixture). However, this is not discussed in the text. Are all your accessions at Q > 0.6? Without a data table, this cannot be verified. Please provide the supplementary data and discuss how many accessions fall into this admixture category. Additionally, Figures 5b and 5c are sufficient; Figure 5a appears redundant.**

**Response:**

1. We sincerely appreciate the reviewer’s careful suggestion. The complete Q-value dataset for all accessions has been added as supplementary material in the revised manuscript to support the association analysis. We have supplemented relevant statistical analysis in the result section: we counted the number of germplasm with Q-value < 0.6 belonging to admixed groups in the text.

2. We appreciate your valuable suggestion. After referring to common practices in published population genetic research, we respectfully request to retain Figure 5a rather than deleting it. LnP(K) and ΔK curves are routinely displayed together in relevant literatures to jointly determine the optimal K value. The plateau trend of the LnP(K) curve provides supplementary evidence to verify grouping reliability and enhances the reproducibility of our results. Therefore, we hope to keep Figure 5a in the manuscript.

**Line 281: It is mathematically and conceptually impossible for the General Linear Model (GLM) and the Mixed Linear Model (MLM) to yield identical p-values for the same 20 loci. The MLM model, by definition, introduces the Kinship matrix (K) as a random effect to control for population stratification, which inherently reduces the statistical power (resulting in higher p-values) and lowers the individual PVE of the markers compared to an uncorrected GLM. The identical metrics reported by the authors strongly suggest either a major data formatting error or that the MLM multi-locus correction was not executed properly in the software. The authors must provide the exact log outputs and original multi model comparison charts to clear this discrepancy.**

**Response:** Thank you for your careful inspection. Same P-values at partial loci between GLM and MLM are statistically reasonable. The kinship correction effect of MLM is minimal for these 20 markers because their trait-associated variation is independent of population structure and relatedness. Most other markers show distinct P-value discrepancies between the two models, consistent with general statistical rules.

**Lines 309–327: This section contains excessive redundancies that hinder the reader's ability to comprehend your claims and conclusions. Reading the phrase "genetic diversity" fifteen times causes the core message to get lost in the text. I recommend introducing the official abbreviation for the index—Shannon-Weaver diversity index (H')—early in the Materials and Methods and within the tables to reduce text density. Crucially, you must clarify that you are discussing a diversity index calculated from phenotypic characteristics, not molecular ones. The current phrasing is highly confusing. Please shorten the paragraph, remove repetitive statements, and rewrite this section using precise terminology. Furthermore, your interpretation is logically flawed: you draw direct conclusions about genetic variation and breeding potential solely from morphological variation. It would be far more logical to move these conclusions to the section where you discuss the molecular-derived indices—gene diversity index (H) and Shannon information index (I). As your own data demonstrates, morphology is heavily influenced even by subtle environmental variations, resulting in different clusters and different marker associations across the two environments.**

**Response:** We appreciate the reviewer’s valuable comments; we have comprehensively revised this paragraph accordingly: the Shannon-Weaver diversity index (H') has been defined in the Materials and Methods and tables for consistent abbreviated usage, all inappropriate mentions of “genetic diversity” in this section have been amended to phenotypic diversity to clearly specify that H' was calculated from phenotypic rather than molecular data, redundant repetitive content has been pruned and the whole text rephrased with concise precise terms.

**Lines 337–360: There is severe confusion, repetition, and data overlap in this section. References 27 and 29 repeat the exact same data points within the citations, and reference 29 is not even related to soybean research. This paragraph requires a complete rewrite.**

**Response:** We appreciate the reviewer’s careful examination. We have fully rewritten this paragraph to delete repeated experimental data and redundant expressions. The identical cited data between Reference 27 and 29 was caused by misplaced citation markers; we have swapped these two citations to correct the referencing error, and replaced the originally irrelevant Reference 29 with a proper soybean-focused publication.

**Lines 361–377: The authors spend considerable effort trying to justify the discrepancies between molecular and phenotypic clustering, as well as the lack of correlation with geographical origin. However, a non-overlapping pattern between neutral molecular markers (SSR) and adaptive phenotypic traits is a widely accepted norm in plant genetics, not an anomaly. More importantly, the authors completely miss the opportunity to explain this phenomenon using their own Population Structure and GLM/MLM data. Please add a discussion regarding how much of the investigated material falls into the genetic admixture (mixed origin) category, and whether these cultivars and lines share a common genetic base. Furthermore, the phrase "unfavorable cultivation conditions" is inappropriate in this context; please justify or remove it. Your locus association data varies heavily between the two environments despite the similar conditions, indicating a substantial Genotype-by Environment interaction—this must be discussed here. Finally, your cluster analysis overlaps with the structure analysis, showing 3 main groups, which indicates a shared genetic background, with the majority of the material falling into a single group. Addressing this will also help you eliminate the superficial remarks in the subsequent lines.**

**Response:** Thank you for your careful inspection. Same P-values at partial loci between GLM and MLM are statistically reasonable. The kinship correction effect of MLM is minimal for these 20 markers because their trait-associated variation is independent of population structure and relatedness. Most other markers show distinct P-value discrepancies between the two models, consistent with general statistical rules.

**On lines 379–380, the authors state that UPGMA clustering is "prone to human-induced biases". UPGMA is a deterministic, distance-based mathematical algorithm that operates without human intervention. The authors must explain what "human biases" they are referring to. On line 381, the authors state that STRUCTURE analysis yields "more distinct phenotypic characteristics of the clusters". This is fundamentally incorrect. STRUCTURE is a genotype-based Bayesian clustering method that uses allele frequencies. Genetic admixture and complex population stratification actually increase the risk of false-positive marker-trait associations. This is precisely why mixed linear models (MLM) using Q (structure) and K (kinship) matrices are required.**

**Response:** We appreciate the reviewer’s professional correction. We have revised the inappropriate descriptions regarding UPGMA and STRUCTURE analysis: we clarified that the potential errors related to UPGMA clustering are derived from manual genotype identification rather than the algorithm itself, and removed the incorrect statement about phenotypic characteristics, while retaining the core meaning and revising the relevant content with precise and standardized scientific terminology.

**Conclusion (Line 430): The authors use the term "genome-wide SSR linkage mapping". This is conceptually incorrect. Linkage mapping strictly requires bi-parental mapping populations to track recombination frequencies. Since this study utilizes a natural population panel of 150 independent cultivars, the correct terminology is Association mapping (or Association analysis). The authors must replace "linkage mapping" with "association mapping" throughout the manuscript to maintain scientific accuracy.**

**Response:** We thank the reviewer for pointing out this terminology mistake. All inappropriate expressions of “linkage mapping” have been fully replaced with “association mapping” across the whole manuscript in accordance with the experimental population type of natural soybean accessions.
