# Supplementary material for: Genetic diversity and yield trait associations in soybean germplasm using SSR markers
Source: PLoS One. 2026 Aug 4;21(8):e0355194. doi: 10.1371/journal.pone.0355194 (PMC13436762; doi:10.1371/journal.pone.0355194)
Supplement: Supplementary file 8 [file pone.0355194.s010.docx]

**Response Letter**

Manuscript ID: 1710481

Dear Editor and Reviewer:

We sincerely appreciate the reviewer’s careful and thorough evaluation of our revised manuscript, as well as the valuable constructive suggestions to further improve this work. We have carefully considered all comments and made the necessary revisions on the cover letter and the manuscript. The following provides detailed response to each of the comments.

**Reviewer #5:**

**Regarding my previous comment about the identical statistical metrics between the GLM and MLM models: To mathematically support their explanation and results regarding the overlapping p-values, I highly recommend that the authors include the corresponding Quantile-Quantile plots (QQ-plots) for both GLM and MLM in the Supplementary Material. Even providing a representative subset of these plots would be highly beneficial. A QQ-plot is the ideal diagnostic tool to perfectly visualize this overlap and will provide the readers with a clear understanding of the minimal kinship correction effect on these specific loci.**

**Response:** We fully agree with this valuable suggestion. We have generated paired QQ-plots for target traits under both models. All these QQ plots are now added as Figure S13-24 in the Supplementary Materials.

**Regarding my comment on Lines 361-377 (Discrepancies in clustering and G×E interaction): It appears that the authors made a technical oversight during the revision process by pasting the exact same response from the previous question, thereby leaving this comment unaddressed. I strongly advise the authors to revisit this point carefully. Addressing the expected discrepancy between neutral SSRs and adaptive traits, interpreting the genetic admixture from their data, and discussing the apparent Genotype-by-Environment (G×E) interaction will greatly enrich their Discussion section and ensure it is fully substantiated by their actual findings.**

**Response:** We apologize sincerely for the careless technical error in the last revision, where we mistakenly reused the previous reply and failed to thoroughly respond to this critical comment. We have completely reworked this section and supplemented in-depth discussions in the revised Discussion.

**Additionally, upon reviewing the newly provided population structure table, I noticed a mathematical inconsistency in the Q-matrix coefficients. For several accessions, such as individuals 141, 133, 124, and 117, the membership probabilities across the genetic groups do not sum up to 1.00 (100%). By definition, the rows of a Q-matrix must sum to exactly one. I kindly request the authors to double-check their source data and correct these formatting or calculation errors to ensure the validity of the structure covariates used in the models.**

**Response:** We thank the reviewers for carefully pointing out calculation errors in the Q-matrix table. The inconsistencies in the total probabilities for samples 141, 133, 124, and 117 were caused by improper rounding of decimals during table formatting; this issue also affected other samples. We have corrected all affected rows, adjusted the number of decimal places retained, and verified that the sum of the subgroup membership probabilities for each specimen in the updated Q-matrix table strictly equals 1.00.
