# Supplementary material for: Genetic diversity and yield trait associations in soybean germplasm using SSR markers
Source: PLoS One. 2026 Aug 4;21(8):e0355194. doi: 10.1371/journal.pone.0355194 (PMC13436762; doi:10.1371/journal.pone.0355194)
Supplement: Peer review history

# Peer review: Decision Letter 0

**DOI:** 10.1371/journal.pone.0355194.r001

**Authors:** Diaa Abd El-Moneim

14 Jan 2026

PONE-D-25-65645

Genetic Diversity and Yield Trait Associations in Soybean Germplasm Using SSR Markers

PLOS One

Dear Dr. Zong,

Thank you for submitting your manuscript to PLOS ONE. After careful consideration, we feel that it has merit but does not fully meet PLOS ONE’s publication criteria as it currently stands. Therefore, we invite you to submit a revised version of the manuscript that addresses the points raised during the review process.

Please submit your revised manuscript by Feb 28 2026 11:59PM. If you will need more time than this to complete your revisions, please reply to this message or contact the journal office at plosone@plos.org. When you're ready to submit your revision, log on to https://www.editorialmanager.com/pone/ and select the 'Submissions Needing Revision' folder to locate your manuscript file.

Please include the following items when submitting your revised manuscript:

- A letter that responds to each point raised by the academic editor and reviewer(s). You should upload this letter as a separate file labeled 'Response to Reviewers'.
- A marked-up copy of your manuscript that highlights changes made to the original version. You should upload this as a separate file labeled 'Revised Manuscript with Track Changes'.
- An unmarked version of your revised paper without tracked changes. You should upload this as a separate file labeled 'Manuscript'.

If you would like to make changes to your financial disclosure, please include your updated statement in your cover letter. Guidelines for resubmitting your figure files are available below the reviewer comments at the end of this letter.

If applicable, we recommend that you deposit your laboratory protocols in protocols.io to enhance the reproducibility of your results. Protocols.io assigns your protocol its own identifier (DOI) so that it can be cited independently in the future. For instructions see: https://journals.plos.org/plosone/s/submission-guidelines#loc-laboratory-protocols. Additionally, PLOS ONE offers an option for publishing peer-reviewed Lab Protocol articles, which describe protocols hosted on protocols.io. Read more information on sharing protocols at https://plos.org/protocols?utm_medium=editorial-email&utm_source=authorletters&utm_campaign=protocols.

We look forward to receiving your revised manuscript.

Kind regards,

Diaa Abd El-Moneim

Academic Editor

PLOS One

Journal Requirements:

When submitting your revision, we need you to address these additional requirements.

1.Please ensure that your manuscript meets PLOS ONE's style requirements, including those for file naming. The PLOS ONE style templates can be found at

https://journals.plos.org/plosone/s/file?id=wjVg/PLOSOne_formatting_sample_main_body.pdf and

https://journals.plos.org/plosone/s/file?id=ba62/PLOSOne_formatting_sample_title_authors_affiliations.pdf

2. PLOS ONE now requires that authors provide the original uncropped and unadjusted images underlying all blot or gel results reported in a submission’s figures or Supporting Information files. This policy and the journal’s other requirements for blot/gel reporting and figure preparation are described in detail at https://journals.plos.org/plosone/s/figures#loc-blot-and-gel-reporting-requirements and https://journals.plos.org/plosone/s/figures#loc-preparing-figures-from-image-files. When you submit your revised manuscript, please ensure that your figures adhere fully to these guidelines and provide the original underlying images for all blot or gel data reported in your submission. See the following link for instructions on providing the original image data: https://journals.plos.org/plosone/s/figures#loc-original-images-for-blots-and-gels.

In your cover letter, please note whether your blot/gel image data are in Supporting Information or posted at a public data repository, provide the repository URL if relevant, and provide specific details as to which raw blot/gel images, if any, are not available. Email us at plosone@plos.org if you have any questions.

3. We note that the grant information you provided in the ‘Funding Information’ and ‘Financial Disclosure’ sections do not match.

When you resubmit, please ensure that you provide the correct grant numbers for the awards you received for your study in the ‘Funding Information’ section.

4. Please note that funding information should not appear in any section or other areas of your manuscript. We will only publish funding information present in the Funding Statement section of the online submission form. Please remove any funding-related text from the manuscript.

5. We note you have included a table to which you do not refer in the text of your manuscript. Please ensure that you refer to Table 1 in your text; if accepted, production will need this reference to link the reader to the Table.

6. Please include captions for your Supporting Information files at the end of your manuscript, and update any in-text citations to match accordingly. Please see our Supporting Information guidelines for more information: http://journals.plos.org/plosone/s/supporting-information.

7. If the reviewer comments include a recommendation to cite specific previously published works, please review and evaluate these publications to determine whether they are relevant and should be cited. There is no requirement to cite these works unless the editor has indicated otherwise.

[Note: HTML markup is below. Please do not edit.]

Reviewer's Responses to Questions

**Comments to the Author**

1. Is the manuscript technically sound, and do the data support the conclusions?

The manuscript must describe a technically sound piece of scientific research with data that supports the conclusions. Experiments must have been conducted rigorously, with appropriate controls, replication, and sample sizes. The conclusions must be drawn appropriately based on the data presented.

Reviewer #1: Partly

Reviewer #2: Partly

Reviewer #3: Yes

Reviewer #4: Yes

**********

2. Has the statistical analysis been performed appropriately and rigorously?

Reviewer #1: Yes

Reviewer #2: Yes

Reviewer #3: Yes

Reviewer #4: Yes

**********

3. Have the authors made all data underlying the findings in their manuscript fully available?

The PLOS Data policy requires authors to make all data underlying the findings described in their manuscript fully available without restriction, with rare exception (please refer to the Data Availability Statement in the manuscript PDF file). The data should be provided as part of the manuscript or its supporting information, or deposited to a public repository. For example, in addition to summary statistics, the data points behind means, medians and variance measures should be available. If there are restrictions on publicly sharing data—e.g. participant privacy or use of data from a third party—those must be specified.

Reviewer #1: Yes

Reviewer #2: No

Reviewer #3: Yes

Reviewer #4: Yes

**********

4. Is the manuscript presented in an intelligible fashion and written in standard English?

PLOS ONE does not copyedit accepted manuscripts, so the language in submitted articles must be clear, correct, and unambiguous. Any typographical or grammatical errors should be corrected at revision, so please note any specific errors here.

Reviewer #1: Yes

Reviewer #2: Yes

Reviewer #3: Yes

Reviewer #4: Yes

**********

5. Review Comments to the Author

Please use the space provided to explain your answers to the questions above. You may also include additional comments for the author, including concerns about dual publication, research ethics, or publication ethics. (Please upload your review as an attachment if it exceeds 20,000 characters)

Reviewer #1: Reviewer report

The manuscript presents a solid analysis of genetic diversity and SSR-based marker-trait associations in soybean germplasm evaluated across two environments. The study is technically sound and the results are clearly presented. However, the novelty is limited, and several methodological issues require clarification and discussion. Addressing these points would improve the rigor and interpretability of the findings.

Comments

1. While this manuscript does a solid job using SSR markers to map soybean genetic diversity, it’s currently missing a clear 'hook.' Since high-density SNP data is now the standard, the authors really need to highlight what makes this specific study unique. What does this research tell us that previous SSR studies missed? Strengthening the Introduction to clearly state this novelty would make the paper much more compelling (Lines 11-31).

2. Since the phenotypic data comes from just the 2024 season, it’s difficult to tell how much these yield traits fluctuate due to the environment. Because yield is so sensitive to outside factors, the results would be much more robust if we could see trait stability over time. At the very least, the authors should be upfront about this limitation in the discussion and explain how it might influence their conclusions (Lines 87-96).

3. The two test sites are geographically close and share similar weather patterns, I’m wondering if they actually provided enough environmental contrast for the study. It would be helpful if the authors could clarify how distinct these sites really are - otherwise, it becomes difficult to accurately interpret the genotype × environment interactions. A bit more detail here would help justify the experimental design. (Lines 87-92).

4. DNA was extracted from pooled leaf samples per accession. While this approach reduces intra-accession variation, it may also mask within-accession heterogeneity. The rationale and potential consequences of this strategy should be discussed. (Lines 97-100).

5. Using 20 SSR markers to characterize 150 accessions provides a relatively thin look at the genome. With such low marker density, there’s a risk that we’re missing a lot of the genetic landscape, which could make the diversity estimates and association results less reliable. The authors should explicitly address how this limited coverage might impact the strength of their conclusions. (Lines 123-129).

6. The transition from the STRUCTURE analysis to the TASSEL MLM needs more detail. Specifically, the authors should explain how they incorporated the Q and K matrices into their model. Providing the exact configuration used will help clarify how they controlled for population stratification and relatedness. (Lines 161-168; 271-273).

7. The authors set the significance threshold at P < 0.05, but they didn't account for multiple testing. When you're looking at several traits across multiple markers, the odds of finding 'significant' hits by pure chance go way up. To make these associations more believable, the authors should either justify why a raw p-value is sufficient here or, better yet, apply a correction like FDR or Bonferroni to filter out potential false positives. (Lines 170-172).

8. Although the analyses suggest a possible relationship between genetic clustering and geographic origin, this aspect is not explored in depth. The authors are encouraged to examine this relationship more explicitly, for example by assessing whether geographic origin meaningfully contributes to the observed population structure or whether extensive admixture weakens this association. Clarifying this point would substantially strengthen the population genetic interpretation. (Lines 231-238).

9. The UPGMA, PCoA, and STRUCTURE analyses reveal largely consistent patterns. To improve readability, the Results section could be streamlined by reducing repetitive descriptions and instead highlighting the complementary insights provided by each analytical approach. (Lines 231-268).

10. To get a better sense of how well the PCoA represents the actual data, it would be really helpful if the authors could include the percentage of variance explained by the first three principal coordinates. Without those numbers, it’s hard for the reader to tell how much of the genetic story is actually being captured by the plot and how much is just 'noise. (Lines 243-251).

11. Most associated markers account for only a small proportion of the phenotypic variation (around 2–6%). A more critical discussion of their biological significance and practical value for breeding would strengthen the interpretation of these results. (Lines 287-296).

12. Several sections of the Discussion repeat similar points about genetic diversity and the effectiveness of SSR markers. Condensing these passages would improve clarity and allow greater focus on interpretation rather than repetition. (Lines 298-358).

13. The authors report moderate values for PIC, gene diversity, and effective allele number, which is a good start. However, to really understand what these numbers mean for this specific population, it would be helpful to see them compared to previous soybean diversity studies. Placing these results in the context of earlier SSR-based research would clarify whether this germplasm is more or less diverse than what's typically seen in the field. (Lines 341-345).

14. The association results would be strengthened by a clearer comparison with previously published soybean GWAS or QTL studies on yield-related traits. (Lines 391-409).

15. While the manuscript reports stable SSR–trait associations, these markers are not linked to known genomic regions or candidate genes. Even a brief discussion placing them in a genomic context would strengthen the study. (Lines 403-420).

16. Terms such as “hundred-grain weight” and “hundred-seed weight” are used interchangeably. Please standardize terminology throughout. (Throughout manuscript).

17. The manuscript is generally well written, but some sentences – especially in the Discussion – are overly long and repetitive. Careful language editing would improve readability.

Reviewer #2: The authors used SSR markers to study genetic diversity and yield trait correlations in soybean germplasm. The study contains no new information. The manuscript needs multiple modifications.

Abstract

• The issue of the study is unavailable.

• Some scored data about the morphological traits should be included

• The abbreviated SSR should be defined in full name

• A brief conclusion should be written at the end of the abstract

Keywords

The words used for the creation of the title should not be used as the keywords

Introduction

• Recent references should be used

• The authors should provide some lines about genetic diversity methods.

• The methods of association between markers and quantitative traits should be stated

• The gap in the research should be highlighted.

• The hypothesis of the study should be determined

• Originality of the work should be added

Materials and Methods

• All methods used should be supported by the references.

• The manufacture of all materials and instruments should be defined

• All abbreviations should be written in full

Results and Discussion

• The significant status of each measurement should be stated

• The details of the results in the tables and figures must be documented.

• All captions should be improved

• The genetic diversity indices should be performed for populations.

• The gene flow and fixation index should be computed

• The discussion is poor and should be improved. There are a few repeated lines. The contributors should analyze the link between all researched features by explaining the minimum and maximum results. The writers should interpret the relationship between each of the parameters being studied.

Conclusions

• Conclusions should include the most important findings. Additional works should be included.

Reviewer #3: The study aims to evaluate the genetic diversity, and marker trait associations between SSR markers and yield related traits in 150 soybean accessions. The study utilized 20 pairs of SSR primers to genotype diverse soybean accessions. The study was conducted across two environmental conditions.

This study has following strengths:

1. The objective “understanding genetic diversity and identifying marker–trait associations in soybean” is clearly stated and highly relevant to breeding programs.

2. The authors specify the number of SSR primers (20), sample size (150 accessions), and the use of two environmental conditions.

3. The inclusion of multiple analytical methods (GLM, MLM, cluster analysis, PCoA, population structure analysis) shows methodological rigor.

4. The study provides detailed quantitative results (e.g., allele numbers, PIC values, polymorphism rates). Identifying 44 markers associated with yield traits and 6 consistently across environments is a strong highlight.

5. The intended application is clearly stated highlighting the practical value of the study.

Following are few suggestions:

1. Twelve key agronomic traits are mentioned in the abstract. Do add some specific details (what they are? Some description related to substantial phenotypic variation?).

2. Line 30: What you mean by “future conservation management” and “scientific breeding”? Can you replace these with some more familiar terms?

3. Line 34: Provide reference related to “biofuel production”.

4. Line 45 to 47: Simplify the statement “Substantial beneficial ……………..”

5. Line 48 and 51: Is using the term “divergent” or “diverse” right here?

6. Line 64: Please provide reference.

7. Line 68: Please provide reference.

8. Line 69: Please use some simple word instead of “corroborated”

9. Line 75: Use some other word instead of “safeguarding”.

10. Line 88 and 90: Please explain why these environments (E1 and E2) were chosen for this study? How these environments are different? Based on environmental factors, do they differ? Instead of using two similar types of environments, wasn’t it better to go for multi-year study?

11. In results and discussion, please emphasize on interpretations. For example, what do the high polymorphism rates imply? How does clustering across environments support your conclusions? A sentence tying the results to broader implications would strengthen the narrative.

Conclusion/Recommendations

1. Overall, the study presents promising findings. The article is concise and well-written.

2. Recommendation: Minor Revision

Reviewer #4: The research article entitled “Genetic Diversity and Yield Trait Associations in Soybean Germplasm Using SSR Markers” is generally well organized, and the research objectives, methodology, and results are presented in a logical sequence. Most sections are understandable, and the figures and tables appropriately support the text. The manuscript has presented valuable data on Chinese soybean germplasm. However, following comments should be addressed to improve the scientific rigor and clarity of the study.

Major Comments:

1. The field study is only for one year with two locations. Multi-year or multi-location data would strengthen study, and authors should acknowledge this limitation.

2. As the study is comprehensive, however there is need to highlight novel markers associations and the special germplasm. The authors should emphasize how their findings extend beyond previously published soybean SSR studies.

3. PVE values for SSR markers are relatively low (2–6%), authors should discuss the implications for breeding and whether these loci are sufficiently informative for MAS. For instance, in table S6, PVE ranges from 3.22% to 3.90% for the trait plant height indicating that each SSR marker explains only a small fraction of variation in plant height. While this is expected for complex quantitative traits, the manuscript does not adequately discuss the limited practical utility of such low-effect markers for marker-assisted selection. This limitation should be explicitly acknowledged.

4. The supplementary tables report nominal P-values (<0.05), but there is no indication that multiple testing correction (e.g., Bonferroni or FDR) was applied. Given the large number of markers–trait tests typically performed, the absence of correction raises concerns about inflated Type I error.

5. Several markers identified by GLM (are not confirmed by MLM, suggesting potential false positives due to population structure effects. Authors should be cautious in reporting such association and they should emphasize MLM-validated markers. e.g., Satt302 and Satt278 Trait: Pods per Plant

6. Needs to recheck Table S1 critically about the total germplasm, 150 0r 148, as some lines such as NF867 and HH705 are repeated. If the accessions are repeated, then write the paper accordingly.

Minor Comments:

• Need to add more columns in Table S1 such as “Type” (e.g., landrace, improved variety), accession numbers, collection years, or source institutions. Such information is often expected in germplasm tables to allow reproducibility and traceability.

• Some germplasm names include spaces, hyphens, or inconsistent capitalization, which needs uniformity which improves professionalism.

• Table S2: Position” is ambiguous. Does it refer to chromosomal position? Need to clarify.

• Table S2: There is a typo in row 5 (Annealing Temperature: 561 ℃), which is clearly impossible. Such errors reduce confidence in the data.

• Needs uniformity, “Sat_385” and “Satt220.” why some use underscores, some do not? Need to re-check the completed paper

• Table S2: Make sure the formatting is consistent (some have extra spaces, e.g., “58.2 ℃”).

• Table S3: Some numeric entries, e.g., Hundred-seed weight in E2 (24.94366197), are reported with excessive decimal places. Needs rounding to 2–3 decimal places in all other tables too

• Table S3: Need to mention the unit of some agronomic trait such plant height, cm? stem diameter, cm or mm? Units should be included for all the traits in other tables too such as S4

• Table S4: The sample IDs (e.g., 38.00, 30.00) are confusing—are these, averages of specific samples? A clear explanation in a footnote is necessary.

• The term “Interpretation rate (%)” should be replaced with “Phenotypic variance explained (PVE in all the relevant tables.

• While two environments were used, the discussion could more explicitly address environment-specific vs. stable associations, particularly for markers detected in both E1 and E2.

• Decimal precision is inconsistent in all the supplementary tables (e.g., P values reported with varying numbers of digits). For instance, in S15, (some have many decimals, e.g., 0.048112905) which is unnecessary; 3–4 decimal places are sufficient.

• Need to write either 100-seed weight or hundred-grain weight

• Needs to highlight markers detected across both environments and models (GLM and MLM) in all the relevant tables

**********

6. PLOS authors have the option to publish the peer review history of their article (what does this mean?). If published, this will include your full peer review and any attached files.

If you choose “no”, your identity will remain anonymous but your review may still be made public.

**Do you want your identity to be public for this peer review?**  For information about this choice, including consent withdrawal, please see our Privacy Policy.

Reviewer #1: **Yes:** Alibek Zatybekov

Reviewer #2: **Yes:** Nawroz Tahir

Reviewer #3: No

Reviewer #4: No

**********

[NOTE: If reviewer comments were submitted as an attachment file, they will be attached to this email and accessible via the submission site. Please log into your account, locate the manuscript record, and check for the action link "View Attachments". If this link does not appear, there are no attachment files.]

To ensure your figures meet our technical requirements, please review our figure guidelines: https://journals.plos.org/plosone/s/figures

You may also use PLOS’s free figure tool, NAAS, to help you prepare publication quality figures: https://journals.plos.org/plosone/s/figures#loc-tools-for-figure-preparation.

NAAS will assess whether your figures meet our technical requirements by comparing each figure against our figure specifications.

Attachment Submitted filename: Reviewer report.docx

# Author comment: Author response to Decision Letter 1

**DOI:** 10.1371/journal.pone.0355194.r002

27 Feb 2026

Response Letter

Manuscript ID: 1710481

Dear Editor and Reviewers:

We appreciate the constructive feedback provided by the editor and reviewers on our manuscript, which has played a crucial role in improving the quality of our work. We have carefully considered all comments and made the necessary revisions on the cover letter and the manuscript. The following provides detailed response to each of the comments.

Reviewer #1:

The manuscript presents a solid analysis of genetic diversity and SSR-based marker-trait associations in soybean germplasm evaluated across two environments. The study is technically sound and the results are clearly presented. However, the novelty is limited, and several methodological issues require clarification and discussion. Addressing these points would improve the rigor and interpretability of the findings.

Comments

1. While this manuscript does a solid job using SSR markers to map soybean genetic diversity, it’s currently missing a clear 'hook.' Since high-density SNP data is now the standard, the authors really need to highlight what makes this specific study unique. What does this research tell us that previous SSR studies missed? Strengthening the Introduction to clearly state this novelty would make the paper much more compelling (Lines 11-31).

Response: We thank the reviewer for the constructive suggestion. We have revised the Abstract (Lines 11-31) to clearly highlight the novelty and unique value of this study. Specifically, we have clarified that while high-density SNP data is now the standard, our SSR-based analysis has its distinct advantages and fills a gap in previous SSR-related studies on soybean genetic diversity. We have also shortened overly long sentences and removed repetitive content in the Abstract to further improve readability and compellingness.

2. Since the phenotypic data comes from just the 2024 season, it’s difficult to tell how much these yield traits fluctuate due to the environment. Because yield is so sensitive to outside factors, the results would be much more robust if we could see trait stability over time. At the very least, the authors should be upfront about this limitation in the discussion and explain how it might influence their conclusions (Lines 87-96).

Response: We thank the reviewer for this valuable comment. We have added a clear statement in the Discussion to acknowledge the limitation of single-year phenotypic data and its potential impact on the conclusions. We have also indicated that multi-year trials will be needed to improve the robustness of the results in future research.

3. The two test sites are geographically close and share similar weather patterns, I’m wondering if they actually provided enough environmental contrast for the study. It would be helpful if the authors could clarify how distinct these sites really are-otherwise, it becomes difficult to accurately interpret the genotype×environment interactions. A bit more detail here would help justify the experimental design. (Lines 87-92).

Response: We thank the reviewer for this valuable comment. We have clarified in the manuscript that the two test sites are geographically close with similar climatic conditions, and we have noted the potential influence on the interpretation of genotype×environment interactions. We have also added a justification and outlook for further experiments with more contrasting environments.

4. DNA was extracted from pooled leaf samples per accession. While this approach reduces intra-accession variation, it may also mask within-accession heterogeneity. The rationale and potential consequences of this strategy should be discussed. (Lines 97-100).

Response: We appreciate the reviewer’s thoughtful comment. In this study, we used bulked leaf tissue per accession for DNA extraction to minimize the impact of random intra-accession variation and ensure representative genotyping for each accession, which is a common and widely accepted practice in genetic diversity and association analyses using germplasm collections. Although this method may mask minor within-accession heterogeneity, it does not affect the core conclusions of genetic diversity, population structure, and marker-trait association analysis at the accession level, which were the primary focuses of this study.

5. Using 20 SSR markers to characterize 150 accessions provides a relatively thin look at the genome. With such low marker density, there’s a risk that we’re missing a lot of the genetic landscape, which could make the diversity estimates and association results less reliable. The authors should explicitly address how this limited coverage might impact the strength of their conclusions. (Lines 123-129).

Response: We appreciate the reviewer’s thoughtful comment. In this study, we selected 20 highly polymorphic SSR markers. Although marker density is lower than that of high-density SNP platforms, SSR markers show high reproducibility and codominant inheritance. They have sufficient discriminatory power for genetic diversity evaluation and are widely accepted in published studies. Limited genome coverage may reduce the resolution of association analysis, but it does not affect the estimation of genetic diversity, population structure, or preliminary screening of associated markers, which are the main objectives of this study. We have added this discussion in the revised manuscript.

6. The transition from the STRUCTURE analysis to the TASSEL MLM needs more detail. Specifically, the authors should explain how they incorporated the Q and K matrices into their model. Providing the exact configuration used will help clarify how they controlled for population stratification and relatedness. (Lines 161-168; 271-273).

Response: We appreciate the reviewer’s constructive comments. We have revised the description of association analysis models. The content has been simplified, and the model formulas have been replaced with clear textual descriptions to improve readability and conciseness.

7. The authors set the significance threshold at P<0.05, but they didn't account for multiple testing. When you're looking at several traits across multiple markers, the odds of finding 'significant' hits by pure chance go way up. To make these associations more believable, the authors should either justify why a raw p-value is sufficient here or, better yet, apply a correction like FDR or Bonferroni to filter out potential false positives. (Lines 170-172).

Response: We greatly appreciate the reviewer’s valuable comment on multiple testing correction, which is critical for ensuring the reliability of association analysis. We fully acknowledge that uncorrected P-values may increase the risk of false positives.

However, given the specific objectives and marker density of this study, we retained the nominal significance threshold of P < 0.05 for the following reasons: Only 20 SSR markers were used in this study, which is far fewer than typical genome-wide marker sets; This study aimed to conduct an exploratory association analysis to identify preliminary candidate markers rather than to draw definitive conclusions; Overly strict correction would lead to an extremely high threshold and greatly increase the risk of false negatives, which would mask potentially meaningful associations.

8. Although the analyses suggest a possible relationship between genetic clustering and geographic origin, this aspect is not explored in depth. The authors are encouraged to examine this relationship more explicitly, for example by assessing whether geographic origin meaningfully contributes to the observed population structure or whether extensive admixture weakens this association. Clarifying this point would substantially strengthen the population genetic interpretation. (Lines 231-238).

Response: We thank the reviewer for this constructive suggestion. We have further discussed the relationship between genetic clustering and geographic origin in the Discussion. We have clarified the contribution of geographic origin to population structure and the influence of genetic admixture, which strengthens the population genetic interpretation of this study.

9. The UPGMA, PCoA, and STRUCTURE analyses reveal largely consistent patterns. To improve readability, the Results section could be streamlined by reducing repetitive descriptions and instead highlighting the complementary insights provided by each analytical approach. (Lines 231-268).

Response: We appreciate the reviewer’s suggestion to streamline the Results section. We have revised the descriptions of UPGMA, PCoA, and STRUCTURE analyses by removing redundant and repetitive content, while keeping the three methods presented separately for clarity. Each method now highlights its unique and complementary role in inferring population structure, making the results more concise and readable.

10. To get a better sense of how well the PCoA represents the actual data, it would be really helpful if the authors could include the percentage of variance explained by the first three principal coordinates. Without those numbers, it’s hard for the reader to tell how much of the genetic story is actually being captured by the plot and how much is just 'noise. (Lines 243-251).

Response: We appreciate the reviewer’s valuable suggestion.Accordingly, we have added the percentage of variance explained by the first three principal coordinates in the figure legend of PCoA (Fig. 4). This allows readers to better evaluate the quality and explanatory power of the PCoA analysis.

11. Most associated markers account for only a small proportion of the phenotypic variation (around 2–6%). A more critical discussion of their biological significance and practical value for breeding would strengthen the interpretation of these results. (Lines 287-296).

Response: We thank the reviewer for this insightful comment.We have added a more in-depth discussion regarding the biological meaning and breeding relevance of the associated markers, especially addressing their relatively small phenotypic variance explained. This additional discussion improves the interpretation of our association results.

12. Several sections of the Discussion repeat similar points about genetic diversity and the effectiveness of SSR markers. Condensing these passages would improve clarity and allow greater focus on interpretation rather than repetition. (Lines 298-358).

Response: Thank you for pointing out the repetitive content in the Discussion. We have carefully condensed the sections discussing genetic diversity and SSR marker effectiveness, removing all redundant descriptions. The revised manuscript now focuses more on the interpretation of our findings rather than repetitive summaries, which has enhanced the overall clarity and readability of this section.

13. The authors report moderate values for PIC, gene diversity, and effective allele number, which is a good start. However, to really understand what these numbers mean for this specific population, it would be helpful to see them compared to previous soybean diversity studies. Placing these results in the context of earlier SSR-based research would clarify whether this germplasm is more or less diverse than what's typically seen in the field. (Lines 341-345).

Response: We thank the reviewer for the helpful comment. We have added comparisons between our PIC, gene diversity, and effective allele number values and those reported in previous soybean SSR studies. This context clarifies the level of genetic diversity in our germplasm relative to previously reported populations.

14. The association results would be strengthened by a clearer comparison with previously published soybean GWAS or QTL studies on yield-related traits. (Lines 391-409).

Response: We appreciate the reviewer’s valuable suggestion regarding the comparison of our association results with previous GWAS and QTL studies. In this study, we used a relatively small number of SSR markers distributed across the genome, rather than a high density genotyping platform typically used in GWAS. Because of the limited marker density and coverage, our results are more suitable for preliminary marker trait association detection instead of direct comparison with high resolution GWAS or QTL results. We have clarified this point in the revised discussion to better highlight the purpose and scope of the present study.

15. While the manuscript reports stable SSR–trait associations, these markers are not linked to known genomic regions or candidate genes. Even a brief discussion placing them in a genomic context would strengthen the study. (Lines 403-420).

Response: We appreciate the reviewer’s constructive comment. In this study, we used a limited set of SSR markers for preliminary association analysis, rather than high-density genotyping platforms employed in conventional GWAS. Due to the low marker density and insufficient genome coverage, we could not accurately anchor the associated signals to specific genomic regions or known candidate genes. We have clarified this limitation and emphasized the preliminary nature of these associated markers in the revised discussion. More precise mapping and gene identification will be carried out in future studies using high-density markers and larger populations.

16. Terms such as “hundred-grain weight” and “hundred-seed weight” are used interchangeably. Please standardize terminology throughout. (Throughout manuscript).

Response: We thank the reviewer for this reminder. We have standardized the trait name 100-seed weight throughout the entire manuscript and removed inconsistent expressions such as hundred-grain weight.

17. The manuscript is generally well written, but some sentences-especially in the Discussion-are overly long and repetitive. Careful language editing would improve readability.

Response: We appreciate the reviewer’s positive comments and constructive suggestion. We have carefully revised the manuscript, especially the Discussion section, by shortening overly long sentences and removing repetitive content. Language editing has been performed throughout to improve clarity and readability.

Reviewer #2:

The authors used SSR markers to study genetic diversity and yield trait correlations in soybean germplasm. The study contains no new information. The manuscript needs multiple modifications.

Abstract

• The issue of the study is unavailable.

• Some scored data about the morphological traits should be included

• The abbreviated SSR should be defined in full name

• A brief conclusion should be written at the end of the abstract

Response:

We thank the reviewer for the constructive comments. We have added a clear research issue in the manuscript, supplemented statistical data of morphological traits, defined the full name of SSR at first use, and added a brief conclusion at the end of the abstract.

Keywords

The words used for the creation of the title should not be used as the keywords

Response:

We have revised the keywords to avoid repetition with the title, and selected more representative and standardized terms.

Introduction

• Recent references should be used

• The authors should provide some lines about genetic diversity methods.

• The methods of association between markers and quantitative traits should be stated

• The gap in the research should be highlighted.

• The hypothesis of the study should be determined

• Originality of the work should be added

Response:

We appreciate the reviewer’s constructive suggestions. We have further optimized the introduction section by supplementing recent relevant references, briefly introducing common methods for genetic diversity detection and marker-trait association analysis of quantitative traits, and emphasizing the existing research gaps. We have also clearly stated the research hypothesis and highlighted the novelty and practical significance of this work.

Materials and Methods

• All methods used should be supported by the references.

• The manufacture of all materials and instruments should be defined

• All abbreviations should be written in full

Response:

We appreciate the reviewer’s comments. Relevant references have been supplemented to support the adopted methods. The sources and manufacturers of key instruments and reagents have been cl

Attachment Submitted filename: Resoponse letter.docx

# Peer review: Decision Letter 1

**DOI:** 10.1371/journal.pone.0355194.r003

**Authors:** Diaa Abd El-Moneim

26 May 2026

<div>PONE-D-25-65645R1

Genetic Diversity and Yield Trait Associations in Soybean Germplasm Using SSR Markers

PLOS One

Dear Dr. Zong,

Thank you for submitting your manuscript to PLOS ONE. After careful consideration, we feel that it has merit but does not fully meet PLOS ONE’s publication criteria as it currently stands. Therefore, we invite you to submit a revised version of the manuscript that addresses the points raised during the review process.

Please submit your revised manuscript by Jul 10 2026 11:59PM. If you will need more time than this to complete your revisions, please reply to this message or contact the journal office at plosone@plos.org. When you're ready to submit your revision, log on to https://www.editorialmanager.com/pone/ and select the 'Submissions Needing Revision' folder to locate your manuscript file.

Please include the following items when submitting your revised manuscript:

- A letter that responds to each point raised by the academic editor and reviewer(s). You should upload this letter as a separate file labeled 'Response to Reviewers'.
- A marked-up copy of your manuscript that highlights changes made to the original version. You should upload this as a separate file labeled 'Revised Manuscript with Track Changes'.
- An unmarked version of your revised paper without tracked changes. You should upload this as a separate file labeled 'Manuscript'.

If you would like to make changes to your financial disclosure, please include your updated statement in your cover letter. Guidelines for resubmitting your figure files are available below the reviewer comments at the end of this letter.

If applicable, we recommend that you deposit your laboratory protocols in protocols.io to enhance the reproducibility of your results. Protocols.io assigns your protocol its own identifier (DOI) so that it can be cited independently in the future. For instructions see: https://journals.plos.org/plosone/s/submission-guidelines#loc-laboratory-protocols. Additionally, PLOS ONE offers an option for publishing peer-reviewed Lab Protocol articles, which describe protocols hosted on protocols.io. Read more information on sharing protocols at https://plos.org/protocols?utm_medium=editorial-email&utm_source=authorletters&utm_campaign=protocols.

As the corresponding author, your ORCID iD is verified in the submission system and will appear in the published article. PLOS supports the use of ORCID, and we encourage all coauthors to register for an ORCID iD and use it as well. Please encourage your coauthors to verify their ORCID iD within the submission system before final acceptance, as unverified ORCID iDs will not appear in the published article. *Only*  the individual author can complete the verification step; PLOS staff *cannot*  verify ORCID iDs on behalf of authors.

We look forward to receiving your revised manuscript.

Kind regards,

Diaa Abd El-Moneim

Academic Editor

PLOS One

Journal Requirements:

If the reviewer comments include a recommendation to cite specific previously published works, please review and evaluate these publications to determine whether they are relevant and should be cited. There is no requirement to cite these works unless the editor has indicated otherwise.

[Note: HTML markup is below. Please do not edit.]

Reviewers' comments:

Reviewer's Responses to Questions

**Comments to the Author**

1. If the authors have adequately addressed your comments raised in a previous round of review and you feel that this manuscript is now acceptable for publication, you may indicate that here to bypass the “Comments to the Author” section, enter your conflict of interest statement in the “Confidential to Editor” section, and submit your "Accept" recommendation.

Reviewer #5: (No Response)

**********

2. Is the manuscript technically sound, and do the data support the conclusions?

The manuscript must describe a technically sound piece of scientific research with data that supports the conclusions. Experiments must have been conducted rigorously, with appropriate controls, replication, and sample sizes. The conclusions must be drawn appropriately based on the data presented.

Reviewer #5: Partly

**********

3. Has the statistical analysis been performed appropriately and rigorously?

Reviewer #5: No

**********

4. Have the authors made all data underlying the findings in their manuscript fully available?

The PLOS Data policy requires authors to make all data underlying the findings described in their manuscript fully available without restriction, with rare exception (please refer to the Data Availability Statement in the manuscript PDF file). The data should be provided as part of the manuscript or its supporting information, or deposited to a public repository. For example, in addition to summary statistics, the data points behind means, medians and variance measures should be available. If there are restrictions on publicly sharing data—e.g. participant privacy or use of data from a third party—those must be specified.

Reviewer #5: No

**********

5. Is the manuscript presented in an intelligible fashion and written in standard English?

PLOS ONE does not copyedit accepted manuscripts, so the language in submitted articles must be clear, correct, and unambiguous. Any typographical or grammatical errors should be corrected at revision, so please note any specific errors here.

Reviewer #5: Yes

**********

6. Review Comments to the Author

Please use the space provided to explain your answers to the questions above. You may also include additional comments for the author, including concerns about dual publication, research ethics, or publication ethics. (Please upload your review as an attachment if it exceeds 20,000 characters)

Reviewer #5: This manuscript presents an interesting study evaluating 150 soybean genotypes using both phenotypic traits and genetic markers across two distinct environmental conditions. The research is highly relevant within the context of crop breeding and aligns well with the economic and agricultural significance of this crop. However, the study also exhibits several flaws, discrepancies, and conceptual inconsistencies that are of fundamental importance to its scientific contribution and overall validity. These critical issues must be thoroughly addressed and resolved before the manuscript can be considered for publication.

Line 81–82: In the supplementary material, I noticed that most loci are not stable across the two environments. I highly recommend removing this specific part of the sentence.

Lines 102–104: The authors state that they collected 3 leaves per plant. However, it is unclear from how many total plants the leaves were sampled. Were they collected from every replication of the samples and across both environments? If so, the total volume of leaf tissue would be far too large for the commercial extraction kit used. Furthermore, how did this fit into a 5 mL tube, and how can the authors guarantee that taking a tiny 30 mg subsample from such a large bulk mass provides a truly representative sample? There is a lack of clarity here that can be easily resolved.

Line 107: Given that you have three plant replications, please specify how the three random plants were selected. I assume three plants from each environment were used for morphological measurements.

Line 122: Please clarify how many samples were used and from which specific part of the plant they were taken.

Line 122: Shannon-Weaver diversity index – There is no reference, formula, or software package cited for this method. It is unclear whether a software tool was used or if it was calculated based on a previously described protocol in literature. Please add a formal reference or the exact formula to ensure clarity and reproducibility.

Line 165: The software version for iTOL is missing.

Line 179: Phenotypic variation explained (R2) – This specific abbreviation appears only in the Materials and Methods section, after which it is abruptly changed to "PVE". This inconsistency must be standardized throughout the manuscript.

Line 142: The text states a fixed annealing temperature of 50°C. However, Supplementary Figure 2 indicates that the annealing temperatures for each primer actually vary from 40.3°C to 60.5°C. Please resolve this discrepancy.

Figure 2: Figure 2 presents data for only 40 individuals. While I fully understand the technical impossibility of running all samples on a single gel for direct comparison, I strongly recommend adding the remaining gels for at least one marker. If not included as a main figure, this data should be provided as supplementary material. This will allow the reader to gain a clear understanding of how the scoring and sizing of alleles were standardized and compared across separate gels.

Line 265: Supplementary material regarding the Q-values is missing. Since these values are directly utilized in the association analysis, providing this data is essential. Looking at Figure 5, it can be inferred that there are accessions with a Q-value < 0.6, which implies a mixed origin (admixture). However, this is not discussed in the text. Are all your accessions at Q > 0.6? Without a data table, this cannot be verified. Please provide the supplementary data and discuss how many accessions fall into this admixture category. Additionally, Figures 5b and 5c are sufficient; Figure 5a appears redundant.

Line 281: It is mathematically and conceptually impossible for the General Linear Model (GLM) and the Mixed Linear Model (MLM) to yield identical p-values for the same 20 loci. The MLM model, by definition, introduces the Kinship matrix (K) as a random effect to control for population stratification, which inherently reduces the statistical power (resulting in higher p-values) and lowers the individual PVE of the markers compared to an uncorrected GLM. The identical metrics reported by the authors strongly suggest either a major data formatting error or that the MLM multi-locus correction was not executed properly in the software. The authors must provide the exact log outputs and original multi-model comparison charts to clear this discrepancy.

Lines 309–327: This section contains excessive redundancies that hinder the reader's ability to comprehend your claims and conclusions. Reading the phrase "genetic diversity" fifteen times causes the core message to get lost in the text. I recommend introducing the official abbreviation for the index—Shannon-Weaver diversity index (H')—early in the Materials and Methods and within the tables to reduce text density. Crucially, you must clarify that you are discussing a diversity index calculated from phenotypic characteristics, not molecular ones. The current phrasing is highly confusing. Please shorten the paragraph, remove repetitive statements, and rewrite this section using precise terminology. Furthermore, your interpretation is logically flawed: you draw direct conclusions about genetic variation and breeding potential solely from morphological variation. It would be far more logical to move these conclusions to the section where you discuss the molecular-derived indices—gene diversity index (H) and Shannon information index (I). As your own data demonstrates, morphology is heavily influenced even by subtle environmental variations, resulting in different clusters and different marker associations across the two environments.

Lines 337–360: There is severe confusion, repetition, and data overlap in this section. References 27 and 29 repeat the exact same data points within the citations, and reference 29 is not even related to soybean research. This paragraph requires a complete rewrite.

Lines 361–377: The authors spend considerable effort trying to justify the discrepancies between molecular and phenotypic clustering, as well as the lack of correlation with geographical origin. However, a non-overlapping pattern between neutral molecular markers (SSR) and adaptive phenotypic traits is a widely accepted norm in plant genetics, not an anomaly. More importantly, the authors completely miss the opportunity to explain this phenomenon using their own Population Structure and GLM/MLM data. Please add a discussion regarding how much of the investigated material falls into the genetic admixture (mixed origin) category, and whether these cultivars and lines share a common genetic base. Furthermore, the phrase "unfavorable cultivation conditions" is inappropriate in this context; please justify or remove it. Your locus association data varies heavily between the two environments despite the similar conditions, indicating a substantial Genotype-by-Environment interaction—this must be discussed here. Finally, your cluster analysis overlaps with the structure analysis, showing 3 main groups, which indicates a shared genetic background, with the majority of the material falling into a single group.

Addressing this will also help you eliminate the superficial remarks in the subsequent lines. On lines 379–380, the authors state that UPGMA clustering is "prone to human-induced biases". UPGMA is a deterministic, distance-based mathematical algorithm that operates without human intervention. The authors must explain what "human biases" they are referring to. On line 381, the authors state that STRUCTURE analysis yields "more distinct phenotypic characteristics of the clusters". This is fundamentally incorrect. STRUCTURE is a genotype-based Bayesian clustering method that uses allele frequencies. Genetic admixture and complex population stratification actually increase the risk of false-positive marker-trait associations. This is precisely why mixed linear models (MLM) using Q (structure) and K (kinship) matrices are required.

Conclusion (Line 430): The authors use the term "genome-wide SSR linkage mapping". This is conceptually incorrect. Linkage mapping strictly requires bi-parental mapping populations to track recombination frequencies. Since this study utilizes a natural population panel of 150 independent cultivars, the correct terminology is Association mapping (or Association analysis). The authors must replace "linkage mapping" with "association mapping" throughout the manuscript to maintain scientific accuracy.

**********

7. PLOS authors have the option to publish the peer review history of their article (what does this mean?). If published, this will include your full peer review and any attached files.

If you choose “no”, your identity will remain anonymous but your review may still be made public.

**Do you want your identity to be public for this peer review?**  For information about this choice, including consent withdrawal, please see our Privacy Policy.

Reviewer #5: No

**********

[NOTE: If reviewer comments were submitted as an attachment file, they will be attached to this email and accessible via the submission site. Please log into your account, locate the manuscript record, and check for the action link "View Attachments". If this link does not appear, there are no attachment files.]

To ensure your figures meet our technical requirements, please review our figure guidelines: https://journals.plos.org/plosone/s/figures

You may also use PLOS’s free figure tool, NAAS, to help you prepare publication quality figures: https://journals.plos.org/plosone/s/figures#loc-tools-for-figure-preparation.

NAAS will assess whether your figures meet our technical requirements by comparing each figure against our figure specifications.

Attachment Submitted filename: Review.docx.pdf

# Author comment: Author response to Decision Letter 2

**DOI:** 10.1371/journal.pone.0355194.r004

7 Jun 2026

Response Letter

Manuscript ID: 1710481

Dear Editor and Reviewer:

We appreciate the constructive feedback provided by the editor and reviewer on our manuscript, which has played a crucial role in improving the quality of our work. We have carefully considered all comments and made the necessary revisions on the cover letter and the manuscript. The following provides detailed response to each of the comments.

Reviewer #5:

This manuscript presents an interesting study evaluating 150 soybean genotypes using both phenotypic traits and genetic markers across two distinct environmental conditions. The research is highly relevant within the context of crop breeding and aligns well with the economic and agricultural significance of this crop. However, the study also exhibits several flaws, discrepancies, and conceptual inconsistencies that are of fundamental importance to its scientific contribution and overall validity. These critical issues must be thoroughly addressed and resolved before the manuscript can be considered for publication.

Comments

Line 81–82: In the supplementary material, I noticed that most loci are not stable across the two environments. I highly recommend removing this specific part of the sentence.

Response: We thank the reviewer for this valuable comment. We have removed the relevant sentence regarding the instability of most loci across two environments in the revised manuscript accordingly.

Lines 102–104: The authors state that they collected 3 leaves per plant. However, it is unclear from how many total plants the leaves were sampled. Were they collected from every replication of the samples and across both environments? If so, the total volume of leaf tissue would be far too large for the commercial extraction kit used. Furthermore, how did this fit into a 5 mL tube, and how can the authors guarantee that taking a tiny 30 mg subsample from such a large bulk mass provides a truly representative sample? There is a lack of clarity here that can be easily resolved.

Response: We greatly appreciate your careful review and constructive suggestions. Corresponding revisions have been made to this section. Given that plant genomes are stable across different environments, we sampled leaves from one single location. After thorough grinding and mixing, random subsampling was performed on the homogeneous material to ensure sample representativeness.

Line 107: Given that you have three plant replications, please specify how the three random plants were selected. I assume three plants from each environment were used for morphological measurements.

Response: Thank you for the comment. We have added relevant details in the revised manuscript. Consistent with your assumption, three individual plants from each environment were used for morphological trait measurements.

Line 122: Please clarify how many samples were used and from which specific part of the plant they were taken.

Response: Thanks for pointing out this omission. We have clarified the information in line 102-105 accordingly. Consistent with the above sampling method, a total of 150 samples were adopted, and they were collected from the leaves of individual plants.

Line 122: Shannon-Weaver diversity index-There is no reference, formula, or software package cited for this method. It is unclear whether a software tool was used or if it was calculated based on a previously described protocol in literature. Please add a formal reference or the exact formula to ensure clarity and reproducibility.

Response: Thank you for this valuable suggestion. We have supplemented the calculation formula of the Shannon-Weaver diversity index in the revised manuscript .

Line 165: The software version for iTOL is missing.

Response: Thank you for the careful check. We have supplemented the exact software version of iTOL in the revised manuscript. Phylogenetic tree visualization was performed using iTOL v7 (https://itol.embl.de).

Line 179: Phenotypic variation explained (R2) – This specific abbreviation appears only in the Materials and Methods section, after which it is abruptly changed to "PVE". This inconsistency must be standardized throughout the manuscript.

Response: Thank you for pointing out this inconsistent abbreviation. We have uniformly replaced “phenotypic variation explained (R²)” with “PVE” throughout the full manuscript to unify the terminology.

Line 142: The text states a fixed annealing temperature of 50°C. However, Supplementary Figure 2 indicates that the annealing temperatures for each primer actually vary from 40.3°C to 60.5°C. Please resolve this discrepancy.

Response: We appreciate the reviewer’s careful inspection. We have revised the description in the manuscript accordingly. Instead of a unified fixed annealing temperature, the annealing temperature of each primer ranged from 40.3 °C to 60.5 °C.

Figure 2: Figure 2 presents data for only 40 individuals. While I fully understand the technical impossibility of running all samples on a single gel for direct comparison, I strongly recommend adding the remaining gels for at least one marker. If not included as a main figure, this data should be provided as supplementary material. This will allow the reader to gain a clear understanding of how the scoring and sizing of alleles were standardized and compared across separate gels.

Response: Thank you for your valuable feedback. Due to objective limitations of the experimental conditions, the original electrophoresis gel images of the remaining individuals cannot be submitted as supplementary material. This polyacrylamide gel electrophoresis experiment was completed in July 2024. At the time of the experiment, the bands were interpreted manually by visual inspection, and images were captured using early-generation imaging equipment; consequently, complete digital copies of the original images were not retained; At the time of the experiment, gel images from only 40 representative individuals were collected and used for Figure 2.

Line 265: Supplementary material regarding the Q-values is missing. Since these values are directly utilized in the association analysis, providing this data is essential. Looking at Figure 5, it can be inferred that there are accessions with a Q-value < 0.6, which implies a mixed origin (admixture). However, this is not discussed in the text. Are all your accessions at Q > 0.6? Without a data table, this cannot be verified. Please provide the supplementary data and discuss how many accessions fall into this admixture category. Additionally, Figures 5b and 5c are sufficient; Figure 5a appears redundant.

Response:

1. We sincerely appreciate the reviewer’s careful suggestion. The complete Q-value dataset for all accessions has been added as supplementary material in the revised manuscript to support the association analysis. We have supplemented relevant statistical analysis in the result section: we counted the number of germplasm with Q-value < 0.6 belonging to admixed groups in the text.

2. We appreciate your valuable suggestion. After referring to common practices in published population genetic research, we respectfully request to retain Figure 5a rather than deleting it. LnP(K) and ΔK curves are routinely displayed together in relevant literatures to jointly determine the optimal K value. The plateau trend of the LnP(K) curve provides supplementary evidence to verify grouping reliability and enhances the reproducibility of our results. Therefore, we hope to keep Figure 5a in the manuscript.

Line 281: It is mathematically and conceptually impossible for the General Linear Model (GLM) and the Mixed Linear Model (MLM) to yield identical p-values for the same 20 loci. The MLM model, by definition, introduces the Kinship matrix (K) as a random effect to control for population stratification, which inherently reduces the statistical power (resulting in higher p-values) and lowers the individual PVE of the markers compared to an uncorrected GLM. The identical metrics reported by the authors strongly suggest either a major data formatting error or that the MLM multi-locus correction was not executed properly in the software. The authors must provide the exact log outputs and original multi model comparison charts to clear this discrepancy.

Response: Thank you for your careful inspection. Same P-values at partial loci between GLM and MLM are statistically reasonable. The kinship correction effect of MLM is minimal for these 20 markers because their trait-associated variation is independent of population structure and relatedness. Most other markers show distinct P-value discrepancies between the two models, consistent with general statistical rules.

Lines 309–327: This section contains excessive redundancies that hinder the reader's ability to comprehend your claims and conclusions. Reading the phrase "genetic diversity" fifteen times causes the core message to get lost in the text. I recommend introducing the official abbreviation for the index—Shannon-Weaver diversity index (H')—early in the Materials and Methods and within the tables to reduce text density. Crucially, you must clarify that you are discussing a diversity index calculated from phenotypic characteristics, not molecular ones. The current phrasing is highly confusing. Please shorten the paragraph, remove repetitive statements, and rewrite this section using precise terminology. Furthermore, your interpretation is logically flawed: you draw direct conclusions about genetic variation and breeding potential solely from morphological variation. It would be far more logical to move these conclusions to the section where you discuss the molecular-derived indices—gene diversity index (H) and Shannon information index (I). As your own data demonstrates, morphology is heavily influenced even by subtle environmental variations, resulting in different clusters and different marker associations across the two environments.

Response: We appreciate the reviewer’s valuable comments; we have comprehensively revised this paragraph accordingly: the Shannon-Weaver diversity index (H') has been defined in the Materials and Methods and tables for consistent abbreviated usage, all inappropriate mentions of “genetic diversity” in this section have been amended to phenotypic diversity to clearly specify that H' was calculated from phenotypic rather than molecular data, redundant repetitive content has been pruned and the whole text rephrased with concise precise terms.

Lines 337–360: There is severe confusion, repetition, and data overlap in this section. References 27 and 29 repeat the exact same data points within the citations, and reference 29 is not even related to soybean research. This paragraph requires a complete rewrite.

Response: We appreciate the reviewer’s careful examination. We have fully rewritten this paragraph to delete repeated experimental data and redundant expressions. The identical cited data between Reference 27 and 29 was caused by misplaced citation markers; we have swapped these two citations to correct the referencing error, and replaced the originally irrelevant Reference 29 with a proper soybean-focused publication.

Lines 361–377: The authors spend considerable effort trying to justify the discrepancies between molecular and phenotypic clustering, as well as the lack of correlation with geographical origin. However, a non-overlapping pattern between neutral molecular markers (SSR) and adaptive phenotypic traits is a widely accepted norm in plant genetics, not an anomaly. More importantly, the authors completely miss the opportunity to explain this phenomenon using their own Population Structure and GLM/MLM data. Please add a discussion regarding how much of the investigated material falls into the genetic admixture (mixed origin) category, and whether these cultivars and lines share a common genetic base. Furthermore, the phrase "unfavorable cultivation conditions" is inappropriate in this context; please justify or remove it. Your locus association data varies heavily between the two environments despite the similar conditions, indicating a substantial Genotype-by Environment interaction—this must be discussed here. Finally, your cluster analysis overlaps with the structure analysis, showing 3 main groups, which indicates a shared genetic background, with the majority of the material falling into a single group. Addressing this will also help you eliminate the superficial remarks in the subsequent lines.

Response: Thank you for your careful inspection. Same P-values at partial loci between GLM and MLM are statistically reasonable. The kinship correction effect of MLM is minimal for these 20 markers because their trait-associated variation is independent of population structure and relatedness. Most other markers show distinct P-value discrepancies between the two models, consistent with general statistical rules.

On lines 379–380, the authors state that UPGMA clustering is "prone to human-induced biases". UPGMA is a deterministic, distance-based mathematical algorithm that operates without human intervention. The authors must explain what "human biases" they are referring to. On line 381, the authors state that STRUCTURE analysis yields "more distinct phenotypic characteristics of the clusters". This is fundamentally incorrect. STRUCTURE is a genotype-based Bayesian clustering method that uses allele frequencies. Genetic admixture and complex population stratification actually increase the risk of false-positive marker-trait associations. This is precisely why mixed linear models (MLM) using Q (structure) and K (kinship) matrices are required.

Response: We appreciate the reviewer’s professional correction. We have revised the inappropriate descriptions regarding UPGMA and STRUCTURE analysis: we clarified that the potential errors related to UPGMA clustering are derived from manual genotype identification rather than the algorithm itself, and removed the incorrect statement about phenotypic characteristics, while retaining the core meaning and revising the relevant content with precise and standardized scientific terminology.

Conclusion (Line 430): The authors use the term "genome-wide SSR linkage mapping". This is conceptually incorrect. Linkage mapping strictly requires bi-parental mapping populations to track recombination frequencies. Since this study utilizes a natural population panel of 150 independent cultivars, the correct terminology is Association mapping (or Association analysis). The authors must replace "linkage mapping" with "association mapping" throughout the manuscript to maintain scientific accuracy.

Response: We thank the reviewer for pointing out this terminology mistake. All inappropriate expressions of “linkage mapping” have been fully replaced with “association mapping” across the whole manuscript in accordance with the experimental population type of natural soybean accessions.

Attachment Submitted filename: Response to Reviewers.docx

# Peer review: Decision Letter 2

**DOI:** 10.1371/journal.pone.0355194.r005

**Authors:** Diaa Abd El-Moneim

1 Jul 2026

<div>PONE-D-25-65645R2

Genetic Diversity and Yield Trait Associations in Soybean Germplasm Using SSR Markers

PLOS One

Dear Dr. Zong,

Thank you for submitting your manuscript to PLOS ONE. After careful consideration, we feel that it has merit but does not fully meet PLOS ONE’s publication criteria as it currently stands. Therefore, we invite you to submit a revised version of the manuscript that addresses the points raised during the review process.

Please submit your revised manuscript by Aug 15 2026 11:59PM. If you will need more time than this to complete your revisions, please reply to this message or contact the journal office at plosone@plos.org. When you're ready to submit your revision, log on to https://www.editorialmanager.com/pone/ and select the 'Submissions Needing Revision' folder to locate your manuscript file.

Please include the following items when submitting your revised manuscript:

- A letter that responds to each point raised by the academic editor and reviewer(s). You should upload this letter as a separate file labeled 'Response to Reviewers'.
- A marked-up copy of your manuscript that highlights changes made to the original version. You should upload this as a separate file labeled 'Revised Manuscript with Track Changes'.
- An unmarked version of your revised paper without tracked changes. You should upload this as a separate file labeled 'Manuscript'.

If you would like to make changes to your financial disclosure, please include your updated statement in your cover letter. Guidelines for resubmitting your figure files are available below the reviewer comments at the end of this letter.

If applicable, we recommend that you deposit your laboratory protocols in protocols.io to enhance the reproducibility of your results. Protocols.io assigns your protocol its own identifier (DOI) so that it can be cited independently in the future. For instructions see: https://journals.plos.org/plosone/s/submission-guidelines#loc-laboratory-protocols. Additionally, PLOS ONE offers an option for publishing peer-reviewed Lab Protocol articles, which describe protocols hosted on protocols.io. Read more information on sharing protocols at https://plos.org/protocols?utm_medium=editorial-email&utm_source=authorletters&utm_campaign=protocols.

As the corresponding author, your ORCID iD is verified in the submission system and will appear in the published article. PLOS supports the use of ORCID, and we encourage all coauthors to register for an ORCID iD and use it as well. Please encourage your coauthors to verify their ORCID iD within the submission system before final acceptance, as unverified ORCID iDs will not appear in the published article. *Only*  the individual author can complete the verification step; PLOS staff *cannot*  verify ORCID iDs on behalf of authors.

We look forward to receiving your revised manuscript.

Kind regards,

Diaa Abd El-Moneim

Academic Editor

PLOS One

Journal Requirements:

1. If the reviewer comments include a recommendation to cite specific previously published works, please review and evaluate these publications to determine whether they are relevant and should be cited. There is no requirement to cite these works unless the editor has indicated otherwise.

2. Please review your reference list to ensure that it is complete and correct. If you have cited papers that have been retracted, please include the rationale for doing so in the manuscript text, or remove these references and replace them with relevant current references. Any changes to the reference list should be mentioned in the rebuttal letter that accompanies your revised manuscript. If you need to cite a retracted article, indicate the article’s retracted status in the References list and also include a citation and full reference for the retraction notice.

[Note: HTML markup is below. Please do not edit.]

Reviewer's Responses to Questions

**Comments to the Author**

1. If the authors have adequately addressed your comments raised in a previous round of review and you feel that this manuscript is now acceptable for publication, you may indicate that here to bypass the “Comments to the Author” section, enter your conflict of interest statement in the “Confidential to Editor” section, and submit your "Accept" recommendation.

Reviewer #5: (No Response)

**********

2. Is the manuscript technically sound, and do the data support the conclusions?

The manuscript must describe a technically sound piece of scientific research with data that supports the conclusions. Experiments must have been conducted rigorously, with appropriate controls, replication, and sample sizes. The conclusions must be drawn appropriately based on the data presented.

Reviewer #5: Partly

**********

3. Has the statistical analysis been performed appropriately and rigorously?

Reviewer #5: No

**********

4. Have the authors made all data underlying the findings in their manuscript fully available?

The PLOS Data policy requires authors to make all data underlying the findings described in their manuscript fully available without restriction, with rare exception (please refer to the Data Availability Statement in the manuscript PDF file). The data should be provided as part of the manuscript or its supporting information, or deposited to a public repository. For example, in addition to summary statistics, the data points behind means, medians and variance measures should be available. If there are restrictions on publicly sharing data—e.g. participant privacy or use of data from a third party—those must be specified.

Reviewer #5: No

**********

5. Is the manuscript presented in an intelligible fashion and written in standard English?

PLOS ONE does not copyedit accepted manuscripts, so the language in submitted articles must be clear, correct, and unambiguous. Any typographical or grammatical errors should be corrected at revision, so please note any specific errors here.

Reviewer #5: Yes

**********

6. Review Comments to the Author

Please use the space provided to explain your answers to the questions above. You may also include additional comments for the author, including concerns about dual publication, research ethics, or publication ethics. (Please upload your review as an attachment if it exceeds 20,000 characters)

Reviewer #5: Reviewer Report (Round 2)The manuscript has been significantly improved, and the authors have correctly addressed most of the recommendations provided. I have only two remaining recommendations at this stage: Regarding my previous comment about the identical statistical metrics between the GLM and MLM models: To mathematically support their explanation and results regarding the overlapping p-values, I highly recommend that the authors include the corresponding Quantile-Quantile plots (QQ-plots) for both GLM and MLM in the Supplementary Material. Even providing a representative subset of these plots would be highly beneficial. A QQ-plot is the ideal diagnostic tool to perfectly visualize this overlap and will provide the readers with a clear understanding of the minimal kinship correction effect on these specific loci.

Regarding my comment on Lines 361–377 (Discrepancies in clustering and G X E interaction): It appears that the authors made a technical oversight during the revision process by pasting the exact same response from the previous question, thereby leaving this comment unaddressed. I strongly advise the authors to revisit this point carefully. Addressing the expected discrepancy between neutral SSRs and adaptive traits, interpreting the genetic admixture from their data, and discussing the apparent Genotype-by-Environment (G X E) interaction will greatly enrich their Discussion section and ensure it is fully substantiated by their actual findings. Additionally, upon reviewing the newly provided population structure table, I noticed a mathematical inconsistency in the Q-matrix coefficients. For several accessions, such as individuals 141, 133, 124, and 117, the membership probabilities across the genetic groups do not sum up to 1.00 (100%). By definition, the rows of a Q-matrix must sum to exactly one. I kindly request the authors to double-check their source data and correct these formatting or calculation errors to ensure the validity of the structure covariates used in the models.

**********

7. PLOS authors have the option to publish the peer review history of their article (what does this mean?). If published, this will include your full peer review and any attached files.

If you choose “no”, your identity will remain anonymous but your review may still be made public.

**Do you want your identity to be public for this peer review?**  For information about this choice, including consent withdrawal, please see our Privacy Policy.

Reviewer #5: No

**********

[NOTE: If reviewer comments were submitted as an attachment file, they will be attached to this email and accessible via the submission site. Please log into your account, locate the manuscript record, and check for the action link "View Attachments". If this link does not appear, there are no attachment files.]

To ensure your figures meet our technical requirements, please review our figure guidelines: https://journals.plos.org/plosone/s/figures

You may also use PLOS’s free figure tool, NAAS, to help you prepare publication quality figures: https://journals.plos.org/plosone/s/figures#loc-tools-for-figure-preparation.

NAAS will assess whether your figures meet our technical requirements by comparing each figure against our figure specifications.

# Author comment: Author response to Decision Letter 3

**DOI:** 10.1371/journal.pone.0355194.r006

11 Jul 2026

Response Letter

Manuscript ID: 1710481

Dear Editor and Reviewer:

We sincerely appreciate the reviewer’s careful and thorough evaluation of our revised manuscript, as well as the valuable constructive suggestions to further improve this work. We have carefully considered all comments and made the necessary revisions on the cover letter and the manuscript. The following provides detailed response to each of the comments.

Reviewer #5:

Regarding my previous comment about the identical statistical metrics between the GLM and MLM models: To mathematically support their explanation and results regarding the overlapping p-values, I highly recommend that the authors include the corresponding Quantile-Quantile plots (QQ-plots) for both GLM and MLM in the Supplementary Material. Even providing a representative subset of these plots would be highly beneficial. A QQ-plot is the ideal diagnostic tool to perfectly visualize this overlap and will provide the readers with a clear understanding of the minimal kinship correction effect on these specific loci.

Response: We fully agree with this valuable suggestion. We have generated paired QQ-plots for target traits under both models. All these QQ plots are now added as Figure S13-24 in the Supplementary Materials.

Regarding my comment on Lines 361-377 (Discrepancies in clustering and G×E interaction): It appears that the authors made a technical oversight during the revision process by pasting the exact same response from the previous question, thereby leaving this comment unaddressed. I strongly advise the authors to revisit this point carefully. Addressing the expected discrepancy between neutral SSRs and adaptive traits, interpreting the genetic admixture from their data, and discussing the apparent Genotype-by-Environment (G×E) interaction will greatly enrich their Discussion section and ensure it is fully substantiated by their actual findings.

Response: We apologize sincerely for the careless technical error in the last revision, where we mistakenly reused the previous reply and failed to thoroughly respond to this critical comment. We have completely reworked this section and supplemented in-depth discussions in the revised Discussion.

Additionally, upon reviewing the newly provided population structure table, I noticed a mathematical inconsistency in the Q-matrix coefficients. For several accessions, such as individuals 141, 133, 124, and 117, the membership probabilities across the genetic groups do not sum up to 1.00 (100%). By definition, the rows of a Q-matrix must sum to exactly one. I kindly request the authors to double-check their source data and correct these formatting or calculation errors to ensure the validity of the structure covariates used in the models.

Response: We thank the reviewers for carefully pointing out calculation errors in the Q-matrix table. The inconsistencies in the total probabilities for samples 141, 133, 124, and 117 were caused by improper rounding of decimals during table formatting; this issue also affected other samples. We have corrected all affected rows, adjusted the number of decimal places retained, and verified that the sum of the subgroup membership probabilities for each specimen in the updated Q-matrix table strictly equals 1.00.

Attachment Submitted filename: Response_to_Reviewers_auresp_3.docx

# Editor report: Decision Letter 3

**DOI:** 10.1371/journal.pone.0355194.r007

**Authors:** Diaa Abd El-Moneim

20 Jul 2026

Genetic Diversity and Yield Trait Associations in Soybean Germplasm Using SSR Markers

PONE-D-25-65645R3

Dear Dr. Zong,

We’re pleased to inform you that your manuscript has been judged scientifically suitable for publication and will be formally accepted for publication once it meets all outstanding technical requirements.

Within one week, you’ll receive an e-mail detailing the required amendments. When these have been addressed, you’ll receive a formal acceptance letter and your manuscript will be scheduled for publication.

An invoice will be generated when your article is formally accepted. Please note, if your institution has a publishing partnership with PLOS and your article meets the relevant criteria, all or part of your publication costs will be covered. Please make sure your user information is up-to-date by logging into Editorial Manager at Editorial Manager® and clicking the ‘Update My Information' link at the top of the page. For questions related to billing, please contact billing support.

If your institution or institutions have a press office, please notify them about your upcoming paper to help maximize its impact. If they’ll be preparing press materials, please inform our press team as soon as possible -- no later than 48 hours after receiving the formal acceptance. Your manuscript will remain under strict press embargo until 2 pm Eastern Time on the date of publication. For more information, please contact onepress@plos.org.

Kind regards,

Diaa Abd El-Moneim

Academic Editor

PLOS One

# Editor report: Acceptance letter

**DOI:** 10.1371/journal.pone.0355194.r008

**Authors:** Diaa Abd El-Moneim

PONE-D-25-65645R3

PLOS One

Dear Dr. Zong,

I'm pleased to inform you that your manuscript has been deemed suitable for publication in PLOS One. Congratulations! Your manuscript is now being handed over to our production team.

At this stage, our production department will prepare your paper for publication. This includes ensuring the following:

* All references, tables, and figures are properly cited

* All relevant supporting information is included in the manuscript submission,

* There are no issues that prevent the paper from being properly typeset

You will receive further instructions from the production team, including instructions on how to review your proof when it is ready. Please keep in mind that we are working through a large volume of accepted articles, so please give us a few days to review your paper and let you know the next and final steps.

Lastly, if your institution or institutions have a press office, please let them know about your upcoming paper now to help maximize its impact. If they'll be preparing press materials, please inform our press team within the next 48 hours. Your manuscript will remain under strict press embargo until 2 pm Eastern Time on the date of publication. For more information, please contact onepress@plos.org.

You will receive an invoice from PLOS for your publication fee after your manuscript has reached the completed accept phase. If you receive an email requesting payment before acceptance or for any other service, this may be a phishing scheme. Learn how to identify phishing emails and protect your accounts at https://explore.plos.org/phishing.

If we can help with anything else, please email us at customercare@plos.org.

Thank you for submitting your work to PLOS ONE and supporting open access.

Kind regards,

PLOS ONE Editorial Office Staff

on behalf of

Dr. Diaa Abd El-Moneim

Academic Editor

PLOS One
